# Maternal causation of early-onset pre-eclampsia: excessive endometrial gland-derived apolipoprotein D induces placental ferroptosis and developmental abnormalities

**DOI:** 10.1186/s12929-025-01199-7

**Published:** 2025-12-10

**Authors:** Yang Dong, Cheuk-Lun Lee, Jianlin Li, Xiaofeng Liu, Qunxiong Zeng, Jiangming Zhong, Qingqing Zhang, Ting Wu, Vivian W. Y. Ng, Calvin K. F. Lee, Graham J. Burton, Ernest H. Y. Ng, William S. B. Yeung, Ka-Wang Cheung, Philip C. N. Chiu

**Affiliations:** 1 Department of Obstetrics and Gynaecology, School of Clinical Medicine, LKS Faculty of Medicine, The University of Hong Kong, Hong Kong, China; 2 The University of Hong Kong Shenzhen Key Laboratory of Fertility Regulation, The University of Hong Kong-Shenzhen Hospital, Shenzhen, China; 3 Department of Health Technology and Informatics, The Hong Kong Polytechnic University, Hong Kong, China; 4 School of Biomedical Sciences, Li Kai Shing Faculty of Medicine, The University of Hong Kong, Hong Kong, China; 5 Department of Obstetrics and Gynaecology, The University of Hong Kong-Shenzhen Hospital, Shenzhen, China; 6 Department of Obstetrics, Jinan University, Guangzhou, China; 7 Department of Physiology, Development and Neuroscience, University of Cambridge, Cambridge, UK

**Keywords:** Apolipoprotein D, Pre-eclampsia, Endometrial organoid, Placenta, Ferroptosis, Early detection

## Abstract

**Background:**

Early-onset pre-eclampsia (ePE) is a severe pregnancy complication characterized by dysregulated trophoblast functions and impaired placentation during early pregnancy, leading to substantial maternal and fetal morbidity. While circumstantial evidence indicates defective secretion from endometrial glands impairs placental development, direct evidence linking maternal glandular dysfunction to ePE pathogenesis remains elusive.

**Methods:**

We established endometrial glandular organoids from women with ePE and healthy pregnancies, analyzing their secretomes by iTRAQ-based proteomics, RNAseq, and spatial transcriptomics. Functional effects of organoid secretomes on trophoblasts were examined in vitro. An endometrial-specific apolipoprotein D (APOD) knock-in mouse model was studied in vivo. APOD levels in first-trimester serum samples from women who later developed ePE were compared to healthy pregnancies.

**Results:**

Secretomes from ePE derived endometrial organoids impeded spiral artery remodeling. Multiomic analyses revealed increased APOD production in both ePE organoids and decidual tissues. APOD overexpression disrupted trophoblast functions and endothelial vascular remodeling in vitro, and recapitulated ePE phenotypes in an APOD knock-in mouse model through PI3K/Akt-mediated placental ferroptosis and potential ER stress induction. Ferroptosis inhibition with Fer-1 rescued placental defects and PE symptoms in APOD knock-in mice. Elevated APOD levels in first-trimester serum samples from women who later developed ePE suggest its potential as an early biomarker.

**Conclusion:**

This study provides the first direct evidence linking dysregulated endometrial gland function to defective placentation and ePE. APOD was identified as a crucial endometrial gland-secreted factor contributing to ePE, suggesting its potential as an early biomarker and therapeutic target.

**Supplementary Information:**

The online version contains supplementary material available at 10.1186/s12929-025-01199-7.

## Background

Pre-eclampsia (PE) is a pregnancy complication occurring after 20 weeks of gestation characterized by new-onset hypertension, proteinuria, or other symptoms such as thrombocytopenia, renal insufficiency, and impaired liver function [1–3]. PE is multifactorial, affects 2–5% of pregnancies worldwide and leads to > 60,000 maternal and > 500,000 infant deaths annually. It is associated with a high incidence of fetal growth restriction (FGR) and short- and long-term maternal and perinatal complications. Therefore, PE places a significant burden on global healthcare systems. PE is broadly classified into early- and late-onset types: early-onset PE (ePE) occurs before 34 weeks of gestation and is the most severe clinical variant. ePE involves deficient extravillous trophoblast (EVT) induced remodeling of the maternal spiral arteries, resulting in placental malperfusion [4]. Evidence of impaired spiral artery remodeling is weaker in late-onset PE, and other maternal and placental factors are thought to be more important [2, 3].

Spiral artery remodeling involves several key processes including trophoblast proliferation, differentiation, invasion, and their integration into the endothelium of the spiral arteries, all of which are necessary for the establishment of a functional placenta [5]. In ePE, EVT invasion of the endometrial interstitial compartment is deficient during the first trimester of pregnancy and spiral artery remodeling is inadequate [6]. Defects within the maternal decidua affecting interactions among EVTs, fetal antigens, endometrial stromal and epithelial cells, and decidual leukocytes (such as uterine NK cells), could also contribute to the impaired remodeling [86–88]. Deficient spiral artery remodeling is associated with a higher incidence of others of the “Great Obstetrical Syndromes” (GOS), including fetal growth restriction, preterm labor, and late spontaneous miscarriage [2, 3].

The endometrial glands are most active during early pregnancy [7], coincident with the spiral artery remodeling process. The developing placenta is exposed to the glandular secretions [8, 9] which may modulate the functions of EVT [10, 11] and endothelial cells [12]. The importance of the glands in early pregnancy is highlighted in mice deficient in an endometrial gland-specific gene, Forkhead Box a2 (FOXA2) [8, 13]. Deletion of the gene in the neonatal uterus results in an aglandular uterus, whereas deletion in adult mice does not affect glandular morphology. In both cases, mice are infertile due to the lack of leukemia inhibitory factor (LIF). Although injection of LIF rescues implantation, these aglandular mice experience abortions on Day 10 of pregnancy, corresponding to the time of placental development in the human 1ˢᵗ trimester. In humans, circumstantial evidence suggests that defective endometrial gland function is a cause of pregnancy loss and PE [8, 14]. For example, both miscarriage and PE are correlated with low levels of major endometrial gland secretory products such as glycodelin A [15–18]. However, direct evidence and mechanistic details are lacking. 

Apolipoprotein D (APOD), a member of the lipocalin superfamily, is a glycosylated protein integral to lipid transport and metabolism [19]. This atypical apolipoprotein is characterized by its extensive tissue distribution and its role in a variety of physiological processes that extend beyond lipid dynamics, such as cellular homeostasis, differentiation, immune modulation, and aging [20–22]. Both the endometrial stromal and epithelial cells express APOD mRNA and protein during the secretory phase [20, 23, 24], associated with the window of implantation [25, 26].

In this study, APOD was identified as a key factor secreted by endometrial glands that mediates ePE. Its expression was differentially upregulated in gland organoids derived from the decidua in cases of ePE compared to normotensive (NT) controls. Notably, overexpression of APOD suppressed the vascular remodeling activity of EVT, leading to abnormal placental development in endometrial-specific APOD knock-in mice. This suppression effect was mediated by ferroptosis induced by inhibition of the PI3K/AKT pathway. Clinically, our preliminary data of raised levels of APOD in first-trimester maternal serum samples from women who develop PE pave the way for future research into the potential use of APOD as an early predictive biomarker.

## Methods

### Human specimens

All human studies were conducted in accordance with the Declaration of Helsinki. The involvement of human subjects in this study was approved by the Medical Research Ethics Committee of The University of Hong Kong-Shenzhen Hospital (Ethic-2019-36) and by the Institutional Review Board of The University of Hong Kong/Hospital Authority Hong Kong West Cluster (UW21-790). The criteria for defining ePE included (1) a blood pressure of 140/90 mmHg or greater after 20 weeks and before 34 weeks of gestation, (2) proteinuria of 300 mg/L or greater, and (3) without previous hypertensive or renal disease history. The endometrial tissues from the basal plate of the delivered placenta were obtained with written consent from ePE and NT patients who had a caesarean section (Table S1).

Non-pregnant endometrial tissues were obtained with written consent from biopsies of patients who had not received hormone therapy in preparation for in vitro fertilization.

Maternal late pregnancy (28–42 weeks of gestation) serum samples were obtained with written consent from PE and NT patients before their caesarean section (Table S4). Maternal early pregnancy phase serum samples were obtained from pregnant women undergoing prenatal diagnosis in early pregnancy (11–13^+6^ weeks of gestation). Maternal bloods (790 samples) surplus to the diagnostic needs were collected from the prenatal diagnosis clinics at Tsan Yuk Hospital. As there was no reliable marker for the prediction of PE in early pregnancy, these samples were stored until delivery when the PE status was known. Among the collected samples, 14 of the maternal serum samples developed PE at delivery (Table S5). We randomly selected 14 of the NT serum samples as the control for later experiments.

### Establishment of human endometrial organoid

Human endometrial organoid cultures were established according to the methodologies described by Turco et al., 2017 [82]. Tissue samples were minced into small pieces and digested with collagenase V (1 mg/mL, Sigma, St. Louis, MO) and DNase 1 (1500 U, Sigma) solution in RPMI 1640 medium (Corning, New York, USA)/10% FBS with gentle shaking at 37 °C for 20–30 min. After sorting the glandular tissues through 100 µm and then 40 µm cell strainers (Corning, New York, USA), the sieves were inverted and the retained glands backwashed with PBS and collected. The gland fragments were then washed with PBS twice, resuspended in ice cold Matrigel at a ratio of 1:20 (Corning, NY, USA) and placed in a 48-well plate with 20–25 µL droplet per well. The Matrigel-cell suspension was allowed to set in a 37 °C CO_2_ incubator for 20–30 min. The Matrigel was overlayed with 250 μL of advanced DMEM/F12 (Life Technologies, Waltham, MA) based Organoid Medium (OM, Table S6) per well and placed in the incubator. Apical-in/basal-out organoids were formed within 5 days and the OM was replaced every 2–3 days.

The organoid cultured were passaged by manual pipetting when the density reached 80–90% confluence every 7–10 days. For sub-culture, the Matrigel-organoids with the culture medium were sucked out, the organoids were repeatedly pipetted in 100 µL of Advanced DMEM/ F12 (Life Technologies, Waltham, MA) medium for 100–150 times and centrifuged at 500 *g* for 3 min. After resuspension in Matrigel, the organoids were placed into a 48-well plate in a ratio of 1:5 for subculture. In this study, the NT and ePE organoid at passage 3–10 were used for experimentation.

### Organoid treatment and collection of endometrial organoid secretome

NT and ePE organoid with a density of 30% per well were seeded into a 48-well plate for 5 days of culture. Methods to simulate endometrium in early pregnancy and the collection of endometrial organoid secretome were according to our previous protocol [11] with modifications. In brief, the organoids were treated with estrogen (E2, 10 mM, Sigma E4389, St Louis, MO), progesterone (P4, 1 µM, Sigma P7556) and human chorionic gonadotropin (hCG, 1 µg/mL, Source Bioscience ABC403, UK) to mimic early pregnancy phase [11, 82]. To collect the organoid secretome (conditioned medium), PE/NT or treated organoids were washed twice with 500 μL of supplement-free blank medium DMEM/F-12. After the washes, the organoids were cultured in 200 µL of supplement-free blank medium for 24 h. The conditioned medium containing the organoid secretome was collected and debris was removed by centrifugation at 5000 *g* for 5 min.

Total protein content of the conditioned medium was measured by the BCA protein assay (Thermo Scientific) and the absorbance was measured at 562 nm with a microplate reader (BioTek Synergy H1). Alternatively, to collect the organoids, cell recovery solutions (Corning, NY, USA) of 200 µL were added to each well to resuspend the organoids from Matrigel. The organoids and solution were mixed and placed on ice for 30 min. The organoids were collected after centrifugation at 1000 *g* for 5 min for further experiments.

### Immunofluorescence staining of organoids and human decidua samples

Endometrial organoids were recovered for Immunofluorescence analysis for the expression of endometrial gland markers. Organoids were first removed from Matrigel by the Cell Recovery Solution and were then washed by PBS, fixed by 4% paraformaldehyde, and further embedded in a 4% agarose/5% gelatin mixture. Decidual samples were fixed with 4% paraformaldehyde and processed together with the agarose/gelatin-embedded organoids for paraffin embedding and cut into 5 µm sections.

For immunostaining, antigen retrieval of the deparaffinized sections of agarose/gelatin-embedde organoid and human decidua (5 µm) was performed by heating them in Target Retrieval Solution (G1219, Servicebio). The sections were then permeabilized using 0.1% X-triton (BS084, Biosharp) and blocked with 2% BSA. Sections were stained overnight at 4 °C with endometrial gland markers: an anti-FOXA2 antibody (1:100, ab108422 Abcam, Cambridge), anti-PAX8 antibody (1:100, ab227707,Abcam, Cambridge) and Isotype (1:100, ab199376, Abcam, Cambridge). To examine the localization and expression of APOD, the sections were stained overnight at 4 °C with an anti-APOD antibody (1:100, PA527386, Thermofisher) (Table S7). The sections were then incubated with corresponding secondary antibodies conjugated to Alexa Fluor 488 (anti Rabbit IgG 1:400, A-21206, Invitrogen) or Alexa Fluor 647 (1:400, A-31573, Invitrogen) for 2 h at 4 °C. Nuclei were stained with DAPI (1:2000, #62248, Thermofisher). The slides were observed under a Zeiss Laser scanning confocal microscope and the staining intensities were quantified using the Image-Pro Plus software (Media Cybernetics, 17 Inc., MD, USA). All primary and secondary antibodies used in this study are listed in Table S7*.*

### Analysis of organoid transcriptome by mRNA sequencing

#### Total RNA extraction

Total RNA was extracted from the decidua tissues and organoids using Trizol reagent (Invitrogen, Carlsbad, CA, USA). Samples were frozen in a 2 mL tube using liquid nitrogen, homogenized in 1.5 mL of TRIZOL reagent for 2 min, and then allowed to rest horizontally for 5 min. After centrifugation at 12000 *g* for 5 min at 4 °C, the supernatant was collected and mixed with 0.3 mL chloroform/isoamyl alcohol (24:1). The mixture was vigorously shaken for 15 s and then centrifuged at 12000 g for 10 min at 4 °C. After centrifugation, the upper aqueous phase was mixed with an equal volume of isopropyl alcohol and was centrifuged at 13600 *g* for 20 min at 4 °C. After deserting the supernatant, the RNA pellet was washed twice with 1 mL of 75% ethanol and air-dried for 5–10 min. RNA samples were dissolved in 25-100μL of DEPC-treated water. Total RNA was qualified and quantified using a NanoDrop (Thermo Fisher Scientific, MA, USA) and Agilent 2100 bioanalyzer (Agilent, CA, US).

#### mRNA library construction

mRNA library construction and sequencing were performed by the BGISEQ-500 platform (Beijing Genomics Institute-Shenzhen, BGI-Shenzhen, China). According to the protocol provided by the company, Oligo (dT)-attached magnetic beads were utilized to purify mRNA. Purified mRNA was fragmented into small pieces with fragment buffer (75 mM KCl, 6 mM MgCl2, 50 mM Tris–HCl, pH 8.3). First-strand cDNA was generated using random hexamer-primed reverse transcription, followed by second-strand cDNA synthesis. Afterward, a-Tailing Mix and RNA Index Adapters were added for end repair. The cDNA fragments were amplified by PCR and the resulting PCR products were purified by Ampure XP Beads. The purified products were dissolved in EB (10 mM Tris–HCl, pH8.5) solution and validated on the Agilent 2100 bioanalyzer for quality control. The double-stranded PCR products from the previous step were heat-denatured and circularized by the splint oligo sequence to generate the final library as single-strand circular DNA. The final library was amplified with phi29 to produce DNA nanoballs (DNB), each containing more than 300 copies of single molecule. The DNBs were then loaded into the patterned nanoarray, and single-end 50 base reads were generated by BGISEQ-500.

#### Bioinformatic analysis of mRNA-seq data

To process raw data obtained from BGI, the quality of sequencing reads was first assessed using fastQC (version 0.11.8). Quality control of raw sequences was performed using fastp (version 0.20.0). Reads whose phred quality <  = Q15 were excluded. The resulting clean reads were aligned to the human reference genome (hg38) using Hisat2 (version 2.1.0). Gene counts were identified using featureCounts (version 1.6.4). The gene-level read count matrix was then imported into the R (version 4.2.1) for differential gene expression analysis. DEseq2 (version 1.38.3), edgeR (version 3.40.2) and limma (version 3.54.2) packages were utilized in this process. Differentially expressed genes (DEGs) from each package were further filtered to retain those with raw a *p*-value < 0.05 and a fold change contrast ≥ 2. Downstream analysis was based on DEG that could be detected by all packages. These DEGs were used to generate heatmaps by applying R package pheatmap (version 1.0.12) with the scaling value of the normalized read count matrix. All potential targets of DEGs were selected for functional analysis by Gene Ontology (GO) and Kyoto Encyclopedia of Genes and Genomes (KEGG) analysis using clusterProfiler (version 4.6.2).

### Spatial transcriptome analysis of full-thickness endometrium

Full-thickness endometrial tissues in the secretory phase were collected, with written consent, from patients with ovarian masses who had not undergone prior treatment. Spatial transcriptome analysis of full-thickness endometrial sections was performed at the Beijing Genomics Institute-Shenzhen using our established protocols [89]. These sections were first adhered to a Stereo-seq chip surface and incubated at 37 °C for 3–5 min. They were then fixed in methanol and stained with nucleic acid dye for imaging. The tissue sections on the chip were permeabilized with 0.1% pepsin and underwent RNA capture using DNA Nanoballs, followed by overnight reverse transcription at 42 °C. After washing, the sections were digested with Tissue Removal buffer, treated with Exonuclease I, and amplified using KAPA HiFi Hotstart Ready Mix. The PCR products were then quantified, fragmented, purified, and used to generate DNA Nanoballs for sequencing on an MGI DNBSEQ-Tx sequencer. The raw sequencing data, obtained in FASTQ format, was processed by matching coordinate identity (CID) sequences to the in situ captured chip coordinates. Low-quality unique molecular identifiers (UMIs) were excluded, and the remaining reads were aligned to the Homo sapiens genome assembly GRCh38 using STAR. High-quality mapped reads were counted and assigned to their respective genes, consolidating UMIs with identical CIDs and gene loci. This data was used to generate a gene expression matrix file, which was then imported into Stereopy for analysis. The resolution of the Stereo-seq technology allowed for precise binning of nanopores, with a bin size of 50 used in this study.

### Analysis of organoid secretome by mass spectrometry

#### Protein extraction and trypsin digestion

Proteomics analysis was performed by the BGI-Shenzhen. To minimize the influence of high-abundance proteins, the ProteoMiner kit (Bio-Rad, Cal. USA) with a hexapeptide ligand library was used according to the manufacturer’s instructions. Dithiothreitol (DTT, 0281-100G, Amresco, USA) was added to the samples at a final concentration of 10 mM, and the samples were further incubated at 56 °C for 1 h. Iodoacetamide (IG125-10G, IAM, Sigma, St. Louis, MO) was then added to the samples at a final concentration of 55 mM, and the samples were kept in a dark room for 45 min. Subsequently, the samples were precipitated by adding 5 volumes of cold acetone and incubating at − 20 °C for 2 h or overnight. After centrifugation at 25000 *g* for 15 min at 4 °C, the supernatants were discarded, the samples were air-dried, mixed with protein lysis buffer (without SDS), and sonicated in an ice bath.

The samples were centrifuged at 25000 *g* for 15 min at 4 °C to collect the supernatant. The protein concentration was determined by the Bradford method. Protein samples (100 µg) were digested with a 1:20 ratio of trypsin (Hualishi Scientific, Beijing, China) at 37 °C for 2 h. The digested peptide solution was desalinated using reversed-phase chromatography (strata TM-X 33 μM Polymeric Reversed-Phase, Phenomenex, Torrance, CA, USA). The samples were freeze-dried until further use.

#### Proteomic analysis by 2D nano-liquid chromatography (2DLC)-tandem mass spectrometry (MS/MS)

The peptide samples were solubilized in 50 μL isopropanol and then centrifuged at low speed after vortex shaking. Next, the peptide samples were dissolved with 0.5 M TEAB and labeled with the corresponding iTRAQ label reagent for 2 h. Liquid phase separations of samples were carried out by Shimazu lc-20ab liquid phase system (Shimadzu, Kyoto, Japan) with a 5 µm, 4.6 × 250 mm Gemini C18 column (Phenomenex, Torrance, CA, USA). The elution peak was monitored at an absorbance of 214 nm and one component was collected every minute. These components were combined based on the chromatographic elution peak chart and then freeze-dried. The dried peptide samples were re-dissolved in mobile phase A (2% Acetonitrile, ACN; 0.1% Formic acid, FA) and separated using UltiMate 3000 ultra-high performance liquid chromatography (UHPLC, Thermo Fisher Scientific, San Jose, CA, USA). The samples were first entered trap column for enrichment and desalination and then were connected in series with a self-assembled C18 column (75 μm internal diameter, 3 μm column > particle size, 25 μm column length) for further separation using an effective gradient at a flow rate of 300 nL/min. The peptides separated in the liquid phase were ionized by nano-electron Spray Ionization and then entered the Q-Exactive HF X series mass spectrometer (Thermo Fisher Scientific, San Jose, CA) for data-dependent acquisition (DDA) mode detection.

#### Bioinformatic analysis of proteomics data

Raw LC–MS data were first converted into MGF files. These MGF files were used for protein identification through a database search (NCBInr, SwissProt and UniProt) using a local Mascot server. Protein quantification was performed by the published pipeline IQuant. This pipeline encompassed various steps such as protein identification, tag impurity correction, data normalization, missing value imputation, protein ratio calculation, statistical analysis and results presentation. Proteins were identified with a false discovery rate (FDR) < 0.01. Differentially expressed proteins (DEPs) were identified by comparing between groups and significant DEPs were determined based on *p*-value < 0.05 and fold change contrast ≥ 1.5. Differential proteins from different groups were then subjected to GO and KEGG analysis as mentioned above.

### Determination of APOD in endometrial organoid secretome and serum by ELISA

This assay employed an antibody pair specific for human APOD coated on a 96-well plate according to the manufacturer’s instruction (#NBP2-69833, Novus Biologicals, Colorado, USA). Secretome collected from PE and NT organoid, NT/PE serum samples (1:200 diluted with provided buffer solution) and APOD standards were added into the wells. APOD was bound to the wells by the immobilized antibody for 2.5 h. The wells were subsequently incubated with biotinylated secondary anti-human APOD antibody for 1 h followed by the coating of HRP-conjugated streptavidin for 45 min. The wells were washed four times with 1 × Wash solution between different steps. The 3,3′,5,5′-Tetramethylbenzidine (TMB) substrate solution was added to the wells and the absorbance was measured at 450 nm.

### Lentivirus infection

APOD lenti-vector design and viral production were obtained from GENECHEM (Shanghai, China). We designed the shRNA plasmid/overexpression vectors, embedded it in HIV lentivirus and then infected organoids, and screened the stable knockdown/ overexpression organoids for subsequent experiments. Meanwhile, to successfully knock down, we designed three shRNA for APOD (Table S8). Knockdown (12.5 × 10^6^ Tu/well) and overexpressed (20.8 × 10^6^ Tu/well) lenti-virus were transfected into 70–80% density/well organoids together with their respective control by Hitrans G-B-2 according to the manufacturer instructions, infection medium was changed with organoid medium at 48 h and infection efficiency was tested at 96 h use qPCR, Western-blot and ELISA. Secretome from the stable knockdown/ overexpression organoids were collect for EVTs viability, invasion, integration and HUVEC tube formation assay.

### Quantitative RT-PCR

Total RNA was isolated using the Cell Total RNA Isolation Kit (RE-03111, Foregene, Chengdu, China): After digestion and lysis with Buffer cRL1, the organoid cells were filtered by DNA-cleaning column to collect cell lysis supernatant and remove genomic DNA in the system. After adding 1.6 times Buffer cRL2, the mixture was transferred to the RNA-only column to be purification. After centrifugation, 500ul Buffer RW1 was used to remove protein, 700ul Buffer RW2 was used to desalt twice, and then empty tube centrifugation was applied to remove the residual ethanol. APOD, GPX4 were evaluated using qRT-PCR analysis (PrimeScript™ RT reagent Kit with gDNA Eraser, RR047A, Takara, Kyoto, Japan; Taq Pro Universal SYBR qPCR Master Mix). The steps for removing genomic DNA are shown in Table S9. The mRNA reverse transcription system is shown in Table S10. β-actin was used as internal controls. Primers and other short nucleotide sequences used in the study are shown in Table S11 and the SYBR Green Real-time PCR amplification reaction system is shown in Table S12. PCR conditions for mRNA amplification were set as 95 °C for 30 s, followed by 40 cycles of 95 °C for 10 s and 60 °C for 30 s. mRNA transcription levels were normalized to endogenous β-actin, and were quantified using the delta-delta CT method.

### Functional assays of spiral artery remodeling functions of EVTs and endothelial cells

#### Human primary EVT isolation and cell culture

Human primary EVT was isolated according to a published protocol [90] with slight modifications. First trimester placental tissues were obtained from surgical terminations of pregnancy from patients in The University of Hong Kong—Shenzhen Hospital with written consent (hkuszh2022043). The placental tissues were washed in ice-cold PBS, blood clots and chorion membranes were removed, and the placental tissues were minced by surgical blades. The minced tissue was further digested with trypsin (Gibco) and DNase (Thermofisher) with gentle shaking at 37 °C for 30 min. The digested mixture was filtered through 100 μm and then 40 μm cell strainers. The cell suspension was washed with PBS twice and then layered on Ficoll^®^ Paque Plus and subjected to gradient centrifugation for 30 min at 720 *g* without braking. The cells at the interface layer were collected, washed with PBS and resuspended in complete DMEM-F12 culture medium. The trophoblasts were then seeded on fibronectin-coated plates overnight for selection and differentiation of EVT by cell adhesion. After that, the purity of enriched EVTs adhered to the plate were analyzed by flow cytometry using HLAG-PE antibody (ab24384, Abcam, Figure S8) The isolated EVT were cultured in complete DMEM-F12 culture medium for further experiments. Human Umbilical Vein Endothelial Cells (HUVECs; C-12200, Promocell, Heidelberg, Germany) were cultured in Endothelial Cell Growth Medium (C-22010, Promocell, Heidelberg, Germany) gelatin (1% gelatin, Sigma)-coated plates.

#### Cell viability analysis by cell counting kit-8 (CCK8) assay

For the experimental setup, EVT/HUVEC cells (5 × 10^3^cells/well) were cultured in 96-well plates with 100µL of organoid conditioned medium for 24 h. Oganoid-conditioned medium were with or without blocking anti-APOD antibody (1:300, ab108191,Abcam, Cambridge). After incubating the cells with 10µL of sterile CCK8 dye (Selleck) for 1, 2, and 3 h at 37 °C, absorbance at 450 nm was measured using a Microplate Reader (Thermo Fisher Scientific, San Jose, CA, USA). Cell viability was calculated using the following formula: (Absorbance of Test − Blank Absorbance)/(Absorbance of Control − Blank Absorbance) × 100%.

#### Tube formation assay

Matrigel (270μL per well) was coated on a 24-well plate and allowed to solidify for 40 min. The tube formation assay was performed by seeding HUVECs (0.5 × 10^5^ cells) onto the Matrigel in the presence of organoid-conditioned medium (mixed in a 1:1 ratio with Endothelial Cell Growth Medium) for 16 h, as previously described [46, 91]. Images of the capillary-like vascular network structures were captured under a light microscope using low magnification (4x). Angiogenesis parameters, the number of junctions, were analyzed using the angiogenesis plugins of ImageJ software (National Institute of Health, USA).

#### Transwell invasion assay

Transwell invasion assay was used to assess cell invasiveness following the manufacturer’s instructions (Corning, CA, USA). EVT cells (0.5 × 10^5^ cells/well) were suspended in 200μL of medium without FBS and seeded into the upper chamber of Transwell inserts (8 μm pore size, Corning, CA, USA) with coated with Matrigel [94]. The lower chamber was filled with organoid-conditioned medium. After incubating for 24 h at 37 °C, noninvasive cells attached to the top side of the inserts were removed. The invasive cells attached to the bottom side of the insert were fixed with 4% paraformaldehyde and stained with 0.1% crystal violet in PBS. Images of the stained cells were captured using a light microscope (Leica, Heidelberg, Germany).

#### uPA assay

Urokinase-type plasminogen activator (uPA) expression was evaluated using qRT-PCR analysis. Primers for uPA was shown in Table S11.

#### Extravillous trophoblast integration assay

To investigate the ability of EVTs to integrate into the vascular endothelial network, an in vitro co-culture model of EVTs and endothelial cells was used as described [46]. HUVECs (0.5 × 10^5^) were labeled with CellTracker Green CMFDA dye (Thermo Fisher Scientific) and allowed to form tube-like structures for 15 h. Subsequently, EVT cells (0.5 × 10^5^) labeled with CellTracker Red CMTPX dye (Thermo Fisher Scientific) were added along with organoid conditioned medium (in a 1:1 ratio) and co-cultured with the pre-formed HUVEC tube network for 10 h. Confocal microscopy was used to capture images of EVT integration, which were subsequently analyzed using Image-Pro Plus software (Media Cybernetics, Rockville Pike, MD). The integration rate was calculated as follows: (Area occupied by integrated EVTs [yellow])/(Area occupied by total EVTs [yellow + red]) × 100%.

### Endometrium-specific APOD overexpression mouse model

#### Animal experiments

The animal experimental protocols were approved by the Animal Ethics Committee of Shenzhen Huarui Model Organisms Biotechnology Co (APS-230316-005-01). Apod f/f Tek-Cr and L-stop-L-Apod flox mice (6–8 weeks of age) were housed in a specific pathogen-free animal room with a 14 h light/10 h dark cycle. Gestational age was determined by monitoring the formation of vaginal plugs, with the presence of a plug counted as embryonic day 0.5 (E0.5).

To generate APOD transgenic mice, a genomic region on chromosome 16 containing the APOD gene was amplified from C57BL/6 DNA and cloned into the Rosa26 donor vector (Shanghai Model Organisms Co.). This vector enables transgene expression to be activated through the deletion of an inhibitory DNA element via Cre-mediated recombination (Fig. 6A). To establish an endometrial-specific APOD knock-in transgenic mouse model, TEK-Cre mice, Tg(Tek-cre)1Ywa (Named as Tie2-cre) were purchased from Shanghai Model Organisms Co. (https://www.jax.org/strain/008863), and its MGI (Mouse Genome Informatics) catalogue and introductions are on https://www.informatics.jax.org/allele/MGI:2450311. Prior studies have detected Cre/Tie2 activity in endometrial glands in human, mice and rats [92, 98–100].

The blood pressure of pregnant female mice at E7.5, 8.5, 9.5, 10.5, 11.5, 12.5, 14.5, E15.5, and 17.5 was non-invasively assessed using the BP-2000 blood pressure analysis system (BIOSEB). This measurement involved placing a detector around the tail of the mice and utilizing transmission photoplethysmography to determine the blood pressure, as previously described [46].

Pregnant mice were sacrificed at E17.5. The placentas and fetus were collected and weighed at E17.5. The tissues were fixed by 4% paraformaldehyde (PFA) in PBS for paraffin embedding. Consecutive 5-μm sections of mouse placentae were visualized by hematoxylin and eosin (H&E) staining and immunofluorescence staining.

#### Estimation of Serum APOD, sFlt-1/ PLGF and Urinary protein/Creatinine in mice

Mouse serum samples were obtained from anticoagulated blood at E17.5 and centrifuged at 1000 *g* for 20 min within 30 min of collection. The levels of mouse Serum APOD (#NBP2-69833, Novus Biologicals, Colorado, USA), *sFlt-1* (MM-45637M1, MEIMIAN, China)/*PLGF* (MM-0090M2, MEIMIAN, China) and Urinary protein (MM-44287M1, MEIMIAN, China)/Creatinine (ED-20532, LunchangshuoBiotech, China) were quantified using ELISA kits following the manufacturer’s instructions.

#### Hematoxylin and eosin (H&E) stain

Sections of mouse placenta (5 µm) were subsequently dewaxed, rehydrated and incubated in Hematoxylin (AG1121, Acmec) for 8 min. After that, the sections were incubated by the Differentiation Fluid (AG1121, Acmec) for 1–5 s, Returned Blue Liquid for 1 min (AG1121, Acmec), Eosin (AG1121, Acmec) for 1 min and dehydrated. The placenta sections through the sagittal midline were chosen for imaging by light microscopy.

#### Immunofluorescence staining of mouse decidua and placenta

For immunostaining, antigen retrieval of the deparaffinized sections of mouse placenta (5 µm) was performed by heating them in Target Retrieval Solution (G1219, Servicebio). The sections were then permeabilized using 0.1% X-triton (BS084, Biosharp) and blocked with 2% BSA. To examine the localization and expression of APOD in decidua, the sections were stained overnight at 4 °C with anti-APOD antibody (1:100, PA527386, Thermofisher). To determine structural defects in the placenta, the sections were stained overnight at 4 °C with Monocarboxylate Transporter 4 (MCT4) (1:100, ab308528, Abcam) and Trophoblast-specific protein alpha (Tpbpa) (1:100, ab320823, Abcam).

To determine the ferroptosis phenomenon in the placenta, the sections were stained overnight at 4 °C with GPX4 (1:100, 30388-1-AP, proteintech). The sections were rinsed several times by PBS and then incubated with corresponding secondary antibodies conjugated to Alexa Fluor 488 (anti Rabbit IgG 1:400, A-21206, Invitrogen) for 2 h at 4 °C. Nuclei were stained with DAPI (1:2000, #62248, Thermofisher). The slides were observed under a Zeiss Laser scanning confocal microscope and the staining intensities were quantified by the Image-Pro Plus software (Media Cybernetics, 17 Inc., MD, USA).

### Study of APOD mediates ferroptosis via PI3K/Akt pathway in PE

#### Ferroptosis inhibitor (Fer-1) intraperitoneal injection in Apod f/f Tek-Cr mouse model

1 mg *Ferroptosis inhibitor (Fer-1)* powder was injected into Apod f/f Tek-Cr mice intraperitoneally at the final concentration of 1 mg/kg body weight at E5.5, 7.5, 9.5, 11.5, 13.5, and E15.5. Pregnant mice were sacrificed at E17.5. The placentas and fetus were collected and weighed at E17.5. The tissue was fixed by 4% paraformaldehyde (PFA) in PBS for paraffin embedding. Consecutive 5-μm sections of mouse placentae were visualized by hematoxylin and eosin (H&E) staining and immunofluorescence staining.

#### Estimation of tissue MDA, SOD in mice

The levels of mouse tissue MDA (S0131S, Beyotime, China), SOD (JM-02672M1, JINGMEI, China) were quantified by ELISA-based methods following the manufacturer’s instructions.

#### Transmission electron microscope (TEM)

Placental tissue samples (approximately 1 mm^3^) were carefully dissected from the labyrinth region and immediately fixed in 2.5% glutaraldehyde buffered with 0.1 M phosphate buffer (pH 7.4) at 4 °C overnight. After fixation, tissues were rinsed three times (15 min each) with 0.1 M phosphate buffer (pH 7.4). Samples were then post-fixed in 1% osmium tetroxide (OsO₄) in 0.1 M phosphate buffer at room temperature for 2 h, followed by three additional 15-min rinses in phosphate buffer. Dehydration was performed at 4 °C through a graded ethanol series (50%, 70%, and 90%; 15–20 min each), in a 1:1 mixture of 90% ethanol and 90% acetone for 15–20 min, and finally in pure 90% acetone for another 15–20 min. Complete dehydration was achieved at room temperature by incubating samples in three changes of absolute acetone (100%, 15–20 min each). Tissues were then infiltrated with EPON 812 resin (SPI-Chem, USA) at room temperature in the following sequence: acetone:EPON resin (2:1) for 3–4 h, acetone:EPON resin (1:2) overnight, and pure EPON resin for 2–3 h at 37 °C. Samples were embedded in fresh EPON resin and polymerized sequentially at 37 °C overnight, 45 °C for 12 h, and 60 °C for 48 h. Ultrathin sections (approximately 70 nm) were cut using an ultramicrotome (Leica EM UC7, Leica Microsystems, Germany), double-stained with 2% uranyl acetate and lead citrate, and examined using a transmission electron microscope (Hitachi HT7800, Hitachi High-Technologies Corporation, Japan) at 80 kV. Representative images of mitochondrial and labyrinth ultrastructure were captured for analysis.

#### Flowcytometry

Tissues were chopped and subjected to enzymatic digestion in Hanks’ Balanced Salt Solution (HBSS) containing 2.5 mg/mL collagenase D (Roche) and 0.1 mg/mL DNase I for 20 min at 37 °C with gentle shaking every 5 min. The supernatants were then passed through 100 mm filters. After washing once with MACS buffer (PBS pH 7.4 plus 2% FCS and 2 mM EDTA), erythrocytes were depleted using ACK lysis buffer, and cells were resuspended in HBSS. To perform BODIPY-C11 staining, cells were resuspended in 100 µL Hanks Balanced Salt Solution (HBSS, Gibco C14175500BT), containing 5 mM BODIPYI 581/591 C11 (5um,1:500, D3861, Thermo Fisher) and incubated for 15 min at 37 °C. Cells were washed and resuspended in 200µL fresh HBSS and analyzed immediately with a flow cytometer (Deflex, Beckman). To detect Fe2 + , cells were stained with FerroOrange (1um, 1:1000, MX4559, MKBio) at 37 °C for 30 min. GMFI was calculated. The data were analyzed using FlowJo version 10.1 software (Tree Star, Ashland, USA).

### Western blot

Protein samples were separated on 10% SDS-PAGE and transferred to PVDF membrane. The membrane was then blocked with 5% non-fat milk and incubated with anti-PI3K/ anti-Phospho-AKT antibody/anti-AKT antibody (1:1000 dilution; Cell Signaling Technology #4249/Cell Signaling Technology #4060/Cell Signaling Technology #4691) (Table S7) and the reference protein anti-β-actin antibody followed by horseradish peroxidase-conjugated against rabbit antibody (1:1000 dilution; 7074 s, Cell Signaling Technology, USA). Blotting signals were revealed with ECL Plus (Beyotime, Shanghai, China).

### Immunofluorescence staining of human placenta samples

For immunostaining, antigen retrieval of the deparaffinized sections of human placenta (5 µm) was performed by heating them in Target Retrieval Solution (G1219, Servicebio). The sections were then permeabilized using 0.1% X-triton (BS084, Biosharp) and blocked with 2% BSA. To determine the ferroptosis phenomenon in the placenta, the sections were stained overnight at 4 °C with GPX4 (1:100, 30388-1-AP, proteintech) (Table S7). The sections were then incubated with corresponding secondary antibodies conjugated to Alexa Fluor 488 (anti Rabbit IgG 1:400, A-21206, Invitrogen) for 2 h at 4 °C. Nuclei were stained with DAPI (1:2000, #62248, Thermofisher). The slides were observed under a Zeiss Laser scanning confocal microscope.

### Data availability

All data are available in the main text or the supplementary materials. All bioinformatic analyses were performed using publicly available software as described in Materials and Methods. The mRNA‑seq data were deposited in the NCBI GEO database (https://www.ncbi.nlm.nih.gov/geo/), under the accession code GSE 287645. The data that support the spatial-seq of endometrial gland have been deposited into NGDC (National Genomics Data Center) with accession number OMIX009055. This paper does not report original code. The software used in this study is described in the aforementioned section and the key resources table in detail. Any additional information required to reanalyze the data reported in this paper is available from the lead contact upon request.

### Quantification and statistical analysis

All experiments reported in this study were replicated using independent samples obtained from different donors. Unless otherwise specified, a minimum of three independent experiments were conducted. The data are presented as mean ± standard deviation (SD). Statistical analyses were performed using SPSS software (version 26.0). Differences were evaluated using one-way analysis of variance (ANOVA) and/or Student’s unpaired t-test. All p-values were two-sided, and a significance level of *p* < 0.05 was considered statistically significant.

## Results

### Establishment of organoids with characteristics of endometrial glands from ePE and NT patients

**Fig. 1:**
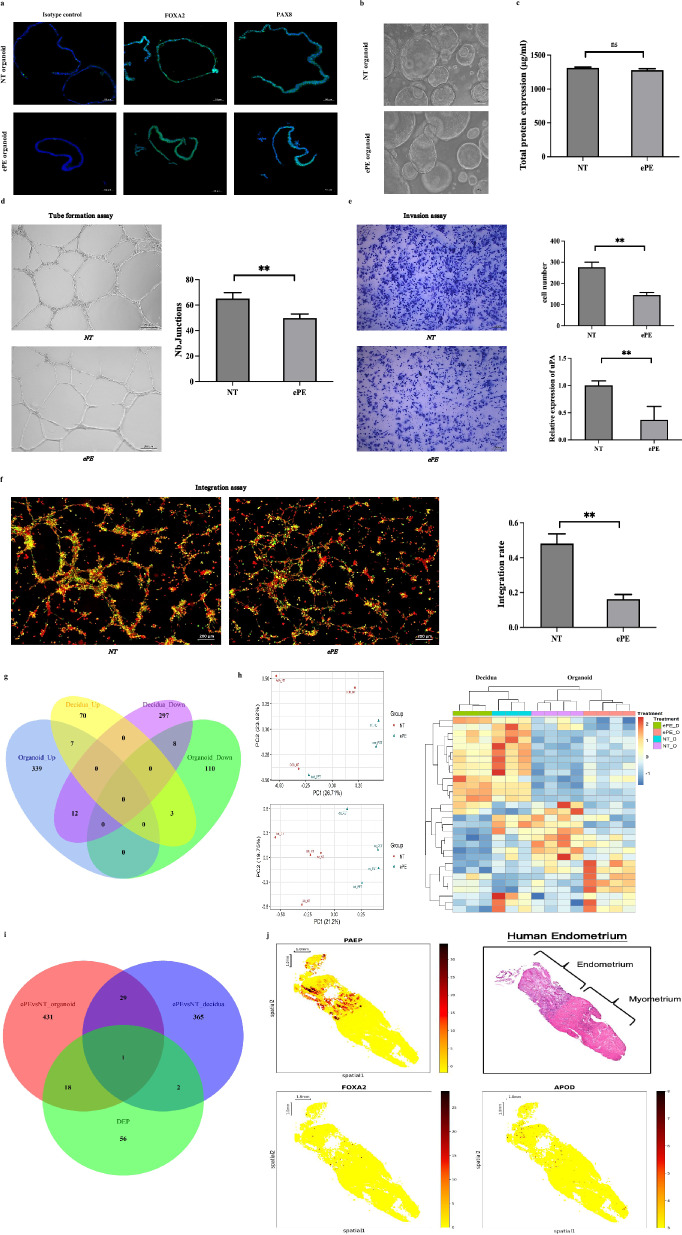
Endometrial organoids from ePE and NT patients show impaired vascular remodeling and distinct molecular profiles. **A** Organoids display morphology and marker expression characteristic of endometrial glands. Immunofluorescence staining shows expressions of endometrial gland markers FOXA2 and PAX8. Scale bar = 50 µm. **B** Representative images demonstrate similari morphology size and quantity between NT and ePE organoids. Scale bar = 200 µm. **C** Comparable protein content in the organoid secretome between NT and ePE organoids. No significant differences were observed. Data are expressed as mean ± SD. N = 3. NS = not significant. **D** ePE organoid secretome inhibits endothelial cell angiogenesis. HUVECs (0.5 × 10^5^) in Matrigel with organoid-conditioned medium and angiogenesis medium (1:1) for 16 h. Scale bar = 200 µm. Data are expressed as mean ± SD. N = 3. **p < 0.01. **E** ePE organoid secretome impedes trophoblast invasion and uPA expression. EVTs (0.5 × 10^5^) in Matrigel-coated Transwell inserts with organoid-conditioned media for invasion assay. uPA expression was measured by qPCR and shown as relative quantification (RQRQ = 2^−ΔΔCT^). Scale bar = 200 µm. Data are expressed as mean ± SD. N = 3. **p < 0.01. **F** ePE organoid secretome reduces EVT integration into endothelial networks. HUVECs (0.5 × 10^5^, labeled green) seeded on Matrigel to form endothelial newtoek followed by EVTs (0.5 × 10^5^, labaeled red) with organoid-conditioned medium for 12 h.. Scale bar = 200 µm. Data are expressed as mean ± SD. N = 3. **p < 0.01. **G** Venn diagram of differentially expressed genes (DEGs) between NT and ePE tissues and organoids. Decidua tissue, N = 3. Organoids, N = 4. **H** Principal Component Analysis (PCA) and hierarchical clustering analysis of DEGs in NT and ePE decidua tissues and organoids. **I** Venn diagram of differentiall expressed proteins (DEPs) in NT and ePE organoids (N = 5), DEG in NT and ePE organoids, and DEG in NT and ePE tissues. **J** Spatial transcriptome profiling of full thickness human endometrium gland showing glandular original of APOD colocalized with endometrial gland-specific marker PAEP

To explore the role of endometrial glandular functions in NT and ePE pregnancies, organoids were generated from glandular fragments obtained from NT (NT organoid) and ePE (ePE organoid) pregnant women at caesarean section (Figure S1). These organoids exhibited the microscopic morphology and recaptured the molecular signatures of endometrial glands, including Forkhead Box A2 (FOXA2) and Paired Box Gene 8 (PAX8) (Fig. 1a). The morphology of the two groups of organoids was similar (Fig. 1b). In this study, NT and ePE organoids at low passages (Passages 3–10) were used to avoid possible change of functions after prolonged culture. Conditioned media were collected from the NT and ePE organoids to investigate the modulatory action of their secretome on processes related to spiral artery remodeling. These media exhibited similar total protein contents (Fig. 1c).

### Secretome from ePE organoid impedes regulatory effects on spiral artery remodeling

We examined events related to spiral artery remodeling using primary EVTs isolated from first-trimester placental tissues and human umbilical vein endothelial cells (HUVECs) (Figure S2). The secretome of ePE organoids, replicated across independent samples from different donors, disrupted the ability of HUVECs to form a tube-like endothelial network compared to the NT organoids, indicating impairement of endothelial behaviour (Fig. 1d). In addition, the ePE organoid secretome reduced EVT invasion by downregulating uPA expression, a key protease involved in extracellular matrix degradation and trophoblast invasion [96] (Fig. 1e). Compared to the NT organoid secretome, the ePE organoid secretome also impaired EVT integration into the endothelial cell network (Fig. 1f). These treatments did not affect viability of HUVECs and EVTs (Figure S3). Overall, the findings suggest that the ePE organoid secretome impairs endothelial cell behaviour and reduces the spiral artery remodeling potential of the trophoblast.

### mRNA and secretome profiling of NT/ePE decidua tissues and organoids

To determine the factors in the secretome of ePE organoids contributing to the EVTs impaired potential for spiral artery remodeling, we performed transcriptomic profiling on human decidual tissue (Table S1) and endometrial organoids obtained from NT and ePE patients. High-throughput RNA-sequencing revealed a total of 397 differentially expressed mRNAs (DEGs) in the primary decidual tissues and 479 DEGs in the organoids. Notably, 15 DEGs were common between the NT/ePE organoids and the decidual tissue comparison (Fig. 1g-h and Table S2). Principal component analysis (PCA) showed distinctive grouping of the samples from different sources (Fig. 1h). The heatmap also clearly demonstrates distinguishable patterns between NT and ePE samples in both the decidual tissues and organoids. It should be noted that while the transcriptomes of organoids differ from those of decidual tissues due to the latter containing a more diverse range of cell types, organoids primarily reflect the characteristics of decidual gland epithelial cells.

Functional analysis of the DEGs was conducted using Kyoto Encyclopedia of Genes and Genomes (KEGG) analysis and Gene Ontology (GO) annotations (Figure S4)*.* DEGs between NT and ePE decidual samples were associated with extracellular matrix organization, reproductive structure/system development, and regulation of angiogenesis. Consistently, DEGs between NT and ePE organoids revealed functional terms specifically related to trophoblast invasiveness, including extracellular structure organization, extracellular matrix organization, cell–matrix adhesion, extracellular matrix structural constituent, cell adhesion molecule binding, metallopeptidase activity, and ECM-receptor interaction. Furthermore, both decidual and organoid DEGs were closely associated with the PI3K-Akt signaling pathway, known for its role in trophoblast invasion and ePE pathogenesis [27, 28].

To investigate the association between ePE glandular secretome and impaired spiral artery remodeling, the secretomes of ePE and NT organoids were analyzed by nano-liquid chromatography-mass spectrometry (n = 5 for each group). Isobaric tags for relative and absolute quantitation (iTRAQ) proteomics analysis revealed a total of 77 differentially expressed proteins (DEPs) between the two groups (Fig. 1i and Table S3). Similar to DEGs, terms associated with extracellular structure, extracellular matrix organization, and extracellular matrix structural constituents were identified in the DEPs. Additional terms included response to oxygen levels, acute inflammatory response, glycosaminoglycan binding, integrin binding, proteasome, complement and coagulation cascades and glycolysis/gluconeogenesis (Figure S5).

Upon matching the DEPs and DEGs, APOD was identified as showing consistent directional changes. Notably, other members of the APO family have also been implicated in PE [29–33]. However, our GO analysis did not reveal enrichment for classical APOD functions, such as transporter activity or cholesterol binding. This suggests that APOD may exert a novel, non-classical effect on trophoblast invasiveness.

Our spatial transcriptome analysis of full-thickness endometrium indicated high expression of APOD in the endometrial glands (Fig. 1j).

### APOD exhibits high expression in ePE endometrial glands and regulates vascular remodeling activities

**Fig. 2:**
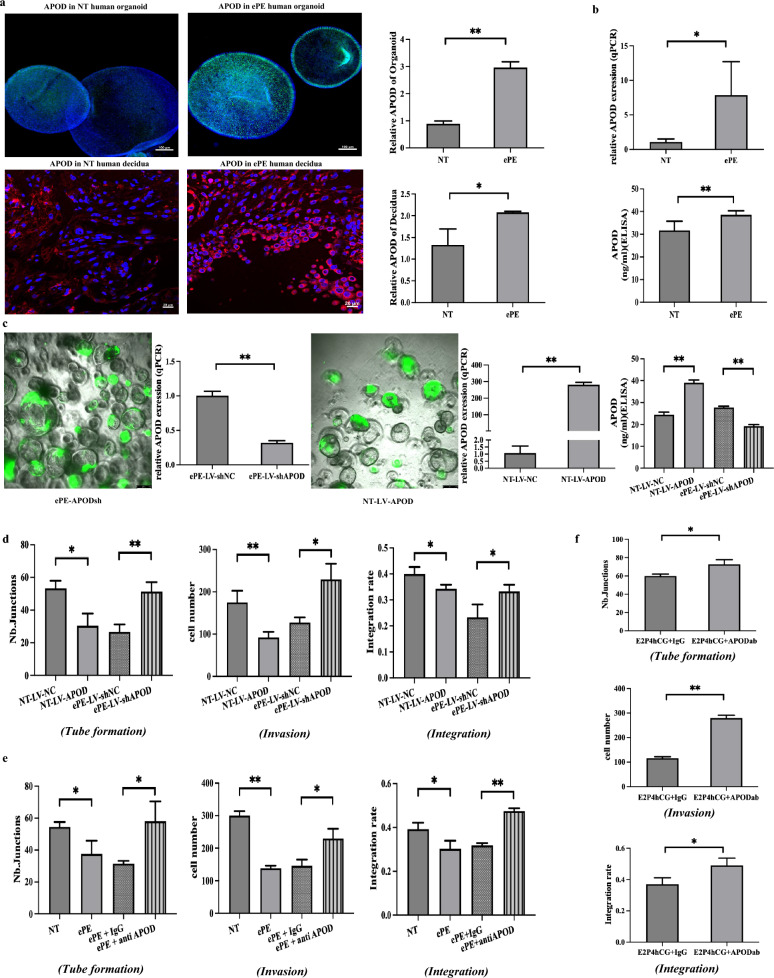
APOD exhibited high expression levels in ePE endometrial gland and regulates spiral artery remodeling. **A** Elevated APOD expression in ePE organoids and decidua compared to that of NT. Immunofluorescence staining and quantification of APOD in organoids (green) and decidua (red). Nuclear counterstain with DAPI. Scale bar = 20/100 µm. N = 3. *p < 0.05, **p < 0.01. **B** Elevated APOD mRNA and protein expression in ePE organoids. mRNA expression was measured by qPCR and shown as relative quantification (RQ = 2^−ΔΔCT^). APOD expression levels in the secretome from ePE and NT organoids were measured by ELISA. N = 5. *p < 0.05, **p < 0.01. **C** APOD levels in the secretions from ePE and NT organoids after lentivirus infection were determined by ELISA. Scale bar = 250 µm. N = 3. **p < 0.01. **D** APOD knockdown secretions from ePE organoids enhances spiral artery remodeling functions: Tube formation assay, invasion assay and integration assay were performed by HUVECs and EVTs with organoid-conditioned medium. Data are expressed as mean ± SD. N = 3. *p < 0.05, **p < 0.01. **E** Blocking APOD with antibodies in ePE enhances spiral artery remodeling functions: Tube formation assay, invasion assay and integration assay were performed by HUVECs and EVTs with ePE, NT organoid-conditioned medium with/without blocking anti-APOD antibody (1:300). N = 3. Data are expressed as mean ± SD. *p < 0.05, **p < 0.01. **F** Blocking APOD with antibodies in early pregnancy phase hormone condition enhances spiral artery remodeling functions: Tube formation assay, invasion assay and integration assay were performed by HUVECs and EVTs with hormone-treated organoid-conditioned media with or without blocking anti-APOD antibody (1:300). Data are expressed as mean ± SD. N = 3. *p < 0.05, **p < 0.01

Our transcriptome/secretome data revealed significantly higher levels of APOD in ePE organoids compared to NT controls. Immunofluoresence analysis confirmed the elevated APOD expression in the ePE endometrial glands and organoids compared to NT samples (Fig. 2a). These findings were further validated through qPCR of organoids and ELISA of organoid secretome (Fig. 2b).

Next, we suppressed APOD expression in ePE organoids and overexpressed APOD in NT organoids using lentivirus technology (Figure S6). The APOD-overexpressing NT organoids (NT-LV-APOD) exhibited higher APOD secretion than the control organoids (NT-LV-NC), while the APOD-suppressed ePE organoids (ePE-LV-shAPOD) showed reduced secretion compared to their corresponding control organoids (ePE-LV-shNC) (Fig. 2c). The secretome of NT-LV-APOD organoids reduced endothelial cell angiogenesis, EVT invasion, and integration, compared to that of the NT-LV-NC control (Fig. 2d). Conversely, the secretome of ePE-LV-shAPOD organoids promoted these activities relative to that of their corresponding control organoids (Fig. 2d). Consistent results were obtained with the use of an APOD function-blocking antibody, which significantly abolished the inhibitory effects of the secretome of ePE organoids on spiral artery remodeling activities compared to the isotype antibody control (Fig. 2e). Neither the lentivirus treatment nor the blocking antibody affected the viability of HUVECs and EVTs (Figure S7-8).

Both the NT and the ePE organoids were established from endometrial tissues obtained at delivery. To verify the significance of our results for placental development, we derived endometrial organoids from non-pregnant endometrial tissues and treated them with hormones to mimic the early pregnancy environment [11]. The organoid secretomes were then collected for functional assays, with and without the use of a function-blocking antibody for APOD. Consistent with the observations using NT/ePE organoids, APOD suppression by function-blocking antibody significantly enhanced the activity of the “early pregnancy” organoid secretome on vascular remodeling activities, including HUVEC angiogenesis, EVT invasion and EVT integration, compared to the control group (Fig. 2f).

### Endometrial-specific APOD knock-in induced ePE-like phenotype in pregnant mice

**Fig. 3:**
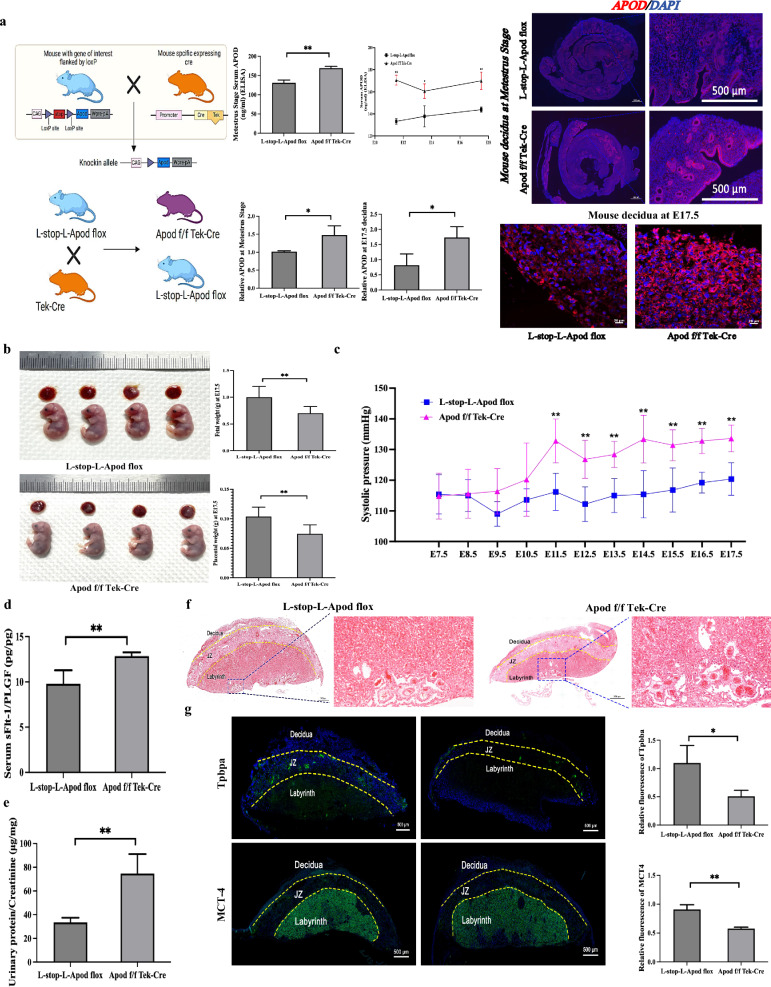
Endometrial-specific APOD knockin induces defective placental development and ePE-like phenotypes in pregnant mice. **A** APOD overexpression increases decidual APOD levels in endometrial-specific knockin mice. Transgenic mice with endometrium-specific knockin APOD were established. Immunofluorescence staining and quantification of APOD expression (red) were performed on decidua from L-stop-L-Apod flox (Control) and Apod f/f Tek-Cr mice at metestrus Stage and at embryonic day E17.5. Nuclei were counterstained with DAPI (blue). Scale bar = 20/500 µm. APOD expression was significantly elevated in Apod f/f Tek-Cre mice compared to controls. APOD expression levels at Metestrus Stage, E11.5, E13.5 and E17.5 were measured by ELISA. Data are expressed as mean ± SD. N = 3. *p < 0.05, **p < 0.01. **B** APOD overexpression reduces placental and fetal weights at E17.5. Placentas were weighed after removal of the umbilical cord and fetal membranes. N = 5 litters. **p < 0.01. **C** APOD overexpression elevates systolic blood pressure during pregnancy. Blood pressure were measured in control and Apod f/f Tek-Cre mice from E7.5 to E17.5. N = 5. **p < 0.01. **D** APOD overexpression increases serum sFlt-1/PLGF ratio at E17.5 measured by ELISA. N = 5. **p < 0.01. **E** APOD overexpression induces proteinuria in pregnant mice. Urinary protein/Creatinine levels of control and Apod f/f Tek-Cr mice at E17.5. N = 5. **p < 0.01. **F** Histological abnormalities observed in placentas of APOD overexpressing mice. Hematoxylin and eosin (H&E) staining of the cross-sections of control and Apod f/f Tek-Cr mice placentas at E17.5 revealed structural abnormalities. Scale bar = 500 µm. N = 3. **G** Altered expression of Tpbpa and MCT4 in placentas of control and Apod f/f Tek-Cr mice. Immunofluorescence staining and quantification of Tpbpa (spongiotrophoblasts and glycogen cells, green) and MCT4 (syncytiotrophoblast layer II, green) expression were performed in mouse placenta. Nuclear counterstain with DAPI (Blue). Perimeters of placental zones (labyrinth; JZ, junctional zone; decidua) were evaluated. Scale bar = 500 µm. N = 3. Data represent mean ± SD. *p < 0.05, ** *p* < 0.01

To investigate the causality of endometrial gland-derived APOD on aberrant decidual and fetal development in vivo, we generated endometrial-specific APOD knock-in transgenic mice. A genomic region on chromosome 16 containing the APOD gene was amplified from C57BL/6 DNA and cloned into the Rosa26 donor vector. TEK-Cre mice were used to establish an endometrial-specific APOD knock-in transgenic mouse model. The Apod f/f Tek-Cre mouse decidua had endometrial glands with normal morphology but they expressed a high level of APOD, in particular in the gland cells at metestrus and E17.5. The serum level of APOD was also elevated in the Apod f/f Tek-Cre mice compared to the L-stop-L-Apod flox mice at metestrus, and at E11.5, E13.5 and E17.5 during pregnancy (Fig. 3a). Endometrium-specific overexpression of APOD resulted in reduced placental and fetal weights at E17.5 without affecting pup number (Fig. 3b and Figure S9). The pregnant mice exhibited increased systolic blood pressure from E11.5 to E17.5 during pregnancy, with no effect on body weight (Fig. 3c and Figure S10). Furthermore, the ratio of serum soluble fms-like tyrosine kinase-1 (*sFlt-1*) to placental growth factor (*PLGF*), an index used to predict PE, was higher in the Apod f/f Tek-Cre mice than the L-stop-L-Apod flox mice at E17.5 (Fig. 3d). Urinary protein/creatinine ratios were also higher in the former group at E17.5 (Fig. 3e).

Histopathological analysis revealed small vessel congestion and necrosis due to arteriolar spasm at E17.5 [34, 35] in the Apod f/f Tek-Cre mice (Fig. 3f). Consistently, the expression of trophoblast-specific protein alpha (TPBPA), and monocarboxylate transporter 4 (MCT4) were also decreased in the Apod f/f Tek-Cre mice (Fig. 3g), suggestive of defective placental development.

Overall, these findings demonstrate that endometrial-specific knock-in of APOD leads to defective placentation and an ePE-like phenotype in mice.

### APOD mediates ferroptosis via PI3K/Akt pathway in ePE placenta

**Fig. 4:**
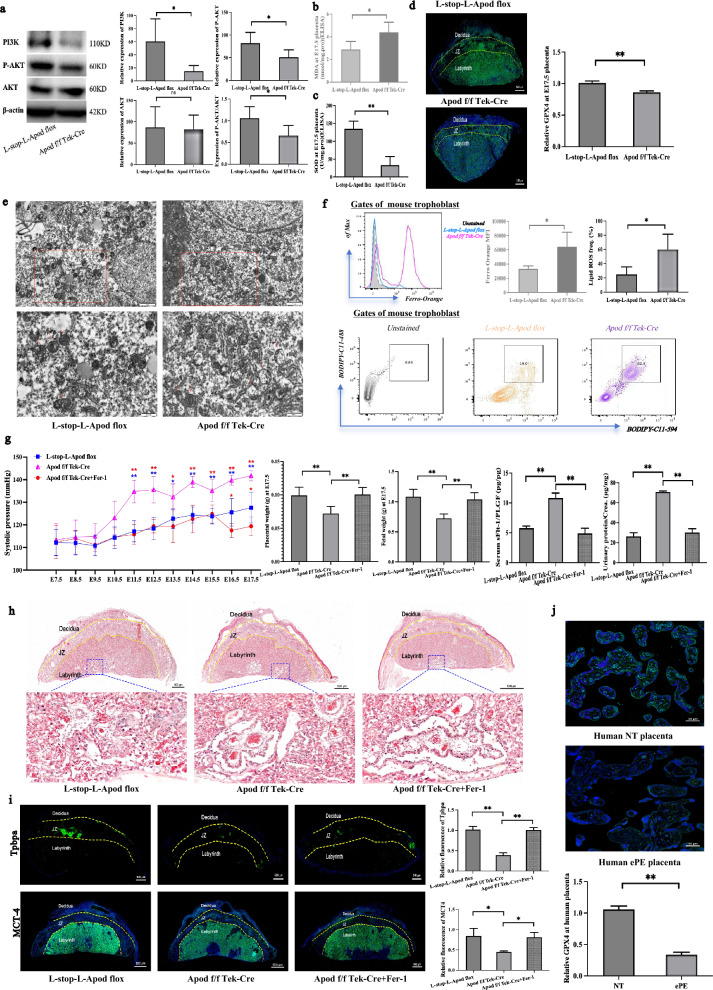
APOD trigger ferroptosis via PI3K/Akt pathway resulting in spiral artery remodeling defects. **A** APOD overexpression inhibits PI3K/Akt pathway in placental tissue. PI3K, Phospho-AKT and AKT expression were assessed from Control and Apod f/f Tek-Cr mice at E17.5. N = 5. *p < 0.05. **B** APOD overexpression increases lipid peroxidation in placental tissue. Lipid peroxifation indicator malondialdehyde (MDA) level were measured by ELISA. N = 5. *p < 0.05. **C** Reduced antioxidant enzyme activity in placentas tissue of APOD overexpressing mice. Superoxide dismutase (SOD) levels were measured by ELISA. N = 5. **p < 0.01. **D** Decreased GPX4 expression indicates ferroptosis. Immunofluorescence staining and quantification of Glutathione Peroxidase 4 (GPX4) expression (green) were performed in placental tissue. Scale bar = 500 µm. N = 3. **p < 0.01. **E** Transmission electron microscopy revealed condensed mitochondrial membranes and reduced mitochondrial cristae in placental tissues of Apod f/f Tek-Cr mice indicating of ferroptosis. Scale bar = 500 nm. N = 3. **F** Increased ferroptosis markers in placentas of APOD overexpressing mice. Flow cytometric quantification of Ferro-Orange (Iron accumulation) and BODIPY-C11-594 (Lipid peroxidation probe) expressions were performed on placental cells. N = 5. *p < 0.05, **p < 0.01. **G** Inhibition of ferroptosis ameliorates ePE-like symptoms in APOD overexpressing mice. Systolic blood pressure (E7.5–17.5, N = 5), fetal and placenta weight (E17.5, N = 5 litters), sFlt-1/PLGF ratio (E17.5, N = 3) and urinary protein/Creatinine ratio (E17.5, N = 3) were measured in control, Apod f/f Tek-Cr and Apod f/f Tek-Cr mice treated with ferroptosis inhibitor Ferrostatin-1 (Fer-1). *p < 0.05, **p < 0.01. **H-I** Inhibition of ferroptosis restored placental architecture abnormalities and expression of Tpbpa and MCT4 in APOD overexpressing mice. H&E staining of placental cross-sections at E17.5 and IFC staining of Tpbpa and MCT4 expression (green) were measured. Scale bar = 500 µm. N = 3. *p < 0.05, **p < 0.01. **J** Decreased of GPX4 expression in human ePE placentas. Immunofluorescence staining and quantification of GPX4 expression (green) were performed in placenta tissue from normotensive (NT) and early pre-eclampsia (ePE) pregnancies. Scale bar = 50 µm. N = 3. **p < 0.01

Our multiomics data suggested that APOD is linked to the PI3K/AKT pathway. The promotion/inhibition of EVT invasion by NT/PE organoid secretions can be reversed by PI3K/AKT pathway inhibitors/activators respectively. Western blot analysis showed that APOD overexpression lowered PI3K, P-AKT, and the phosphorylated AKT (P-AKT)/AKT ratio in the placenta of the Apod f/f Tek-Cre mice (Fig. 4a and Figure S11). Inhibition of PI3K/AKT signaling is associated with ferroptosis [36, 37]. Therefore, we verified the presence of APOD-induced ferroptosis in the placenta of our endometrial-specific APOD knock-in mouse model. Ferroptosis is characterized by high levels of malondialdehyde (MDA) and low levels of superoxide dismutase (SOD). In the Apod f/f Tek-Cr mice, the placental MDA levels were higher (Fig. 4b) and SOD levels were lower (Fig. 4c) when compared with the L-stop-L-Apod flox mice. The level of glutathione peroxidase 4 (GPX4), a key protective factor of ferroptosis, was reduced when ferroptosis occurred. Immunofluorescence staining showed decreased GPX4 levels in the Apod f/f Tek-Cr mouse placenta compared with that of the L-stop-L-Apod flox mice (Fig. 4d). Transmission electron microscopy revealed key characteristics of ferroptosis in the Apod f/f Tek-Cr mouse placenta, including increased mitochondrial membrane density and reduced or absent cristae (Fig. 4e). Flow cytometry showed elevated Fe^2+^ and reactive oxygen species (ROS) in the placenta of the Apod f/f Tek-Cr mice compared to the L-stop-L-Apod flox (Fig. 4f).

To elucidate whether inhibition of ferroptosis could be a treatment option for ePE, ferroptosis inhibitor (Fer-1) was administered intraperitoneally to the Apod f/f Tek-Cre mice. The treatment abolished APOD-induced ferroptosis and improved the ePE-like symptoms relating to blood pressure, placental and fetal weights, sFlt-1/PLGF ratio and protein/creatinine ratio (Fig. 4g). Histopathological analysis showed reduced vascular congestion and necrosis by arteriolar spasm (Fig. 4h). Placenta levels of TPBPA and MCT4 were also increased in the Apod f/f Tek-Cre mice after Fer-1 treatment (Fig. 4i), suggesting improved development. These findings suggest that APOD may mediate ferroptosis via the PI3K/Akt pathway in the ePE placenta. Imortantly, the level of GPX4 was also reduced in human ePE placentas, suggestive of the occurrence of ferroptosis (Fig. 4j).

### Increased circulating APOD levels as an early predictive marker of ePE

**Fig. 5:**
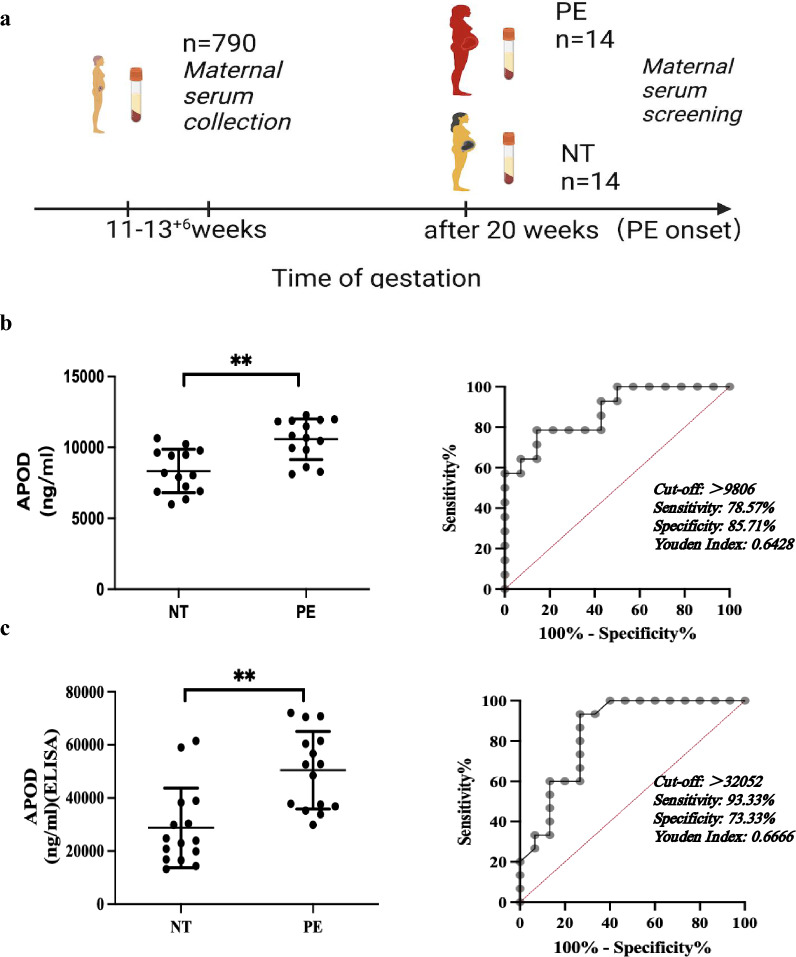
Maternal serum APOD enables early prediction of PE development. **A** Schematic diagram of serum collection from PE and NT pregnancies. **B** Elevated APOD levels in early pregnancy serum samples predict PE. Maternal serum samples obtained obtained from pregnant women undergoing prenatal diagnosis in early pregnancy (11–13^+6^ weeks of gestation). APOD levels in the serum from were measured by ELISA. ROC curve analysis shows diagnostic performance for early PE prediction. N = 14. **p < 0.01. **C** Increased APOD levels in late pregnancy serum of PE patients. APOD levels in the serum from NT and PE patients at late pregnancy phase (28–42 weeks of gestation) were measured by ELISA. ROC curve analysis shows diagnostic performance for establish PE. N = 15. **p < 0.01

To investigate whether APOD levels could predict PE in early pregnancy, we collected peripheral serum samples from 790 pregnant women undergoing prenatal diagnosis in early pregnancy (11–13^+6^ weeks of gestation) and monitored their pregnancy outcomes. These samples were stored until delivery when the PE status was known. Fourteen of these women subsequently developed PE and showed PE symptoms during late pregnancy. These PE serum samples were compared with serum samples from 14 NT pregnant women in the same study cohort. Consistent with the results from decidual tissues and endometrial organoids, the APOD level was increased in the early maternal serum of patients who developed PE later in pregnancy (Fig. 5a-b and Table S4). From the ROC curve, a cut of of 9806 ng/mL will achieve a maximum Youden Index of 0.64 with sensitivity of 78.6% and specificity of 85.7%. Fifteen sera sample were also obtained with written consent from PE and NT women in late pregnancy (28–42 weeks of gestation) before their caesarean section (Table S5). APOD level in serum were determined by ELISA. Consistently, the APOD level was increased in the serum of PE patients in late pregnancy (Fig. 5c). From the ROC curve, a cut of of 32,052 ng/mL will achieve a maximum Youden Index of 0.67 with sensitivity of 93.3% and specificity of 73.3%.

## Discussion

ePE is a leading cause of maternal and fetal morbidity and mortality worldwide [38], yet its underlying mechanisms remain incompletely understood due to the complex pathophysiology. Emerging evidence suggests that dysregulated endometrial gland function may play a crucial role in the pathogenesis of ePE [3, 39]. Using endometrium glandular organoids from ePE patients, this study identifies APOD as a key endometrial gland-secreted factor dysregulated in ePE. Elevated APOD impairs endothelial function and EVT invasiveness, which contribute to impaired spiral artery remodelling, and induces placental ferroptosis via PI3K/AKT signaling, establishing a maternal contribution to ePE pathogenesis. Clinically, early elevation of serum APOD in ePE patients suggesting its potential as a biomarker for early detection of ePE.

Endometrial glands are essential for the establishment of pregnancy, especially during blastocyst implantation and placentation [40]. Studies using ovine endometrial gland knockout (UGKO) model and mutant progesterone-induced UGKO mice have underscored the primary importance of endometrial gland secretions in implantation, endometrial receptivity and first-trimester placental development prior to the establishment of the fetal-maternal interface [8, 41]. Abnormalities in endometrial gland functions are among the major causes of recurrent pregnancy loss [42], PE [3], and various perinatal complications [43].

APOD, a 25 to 30 kDa glycosylated protein, is expressed in the endometrium during the secretory phase, and its expression increases after hormonal (E2 + P4 + hCG) stimulation in vitro [20]. It is also elevated in a consensus endometrial receptivity transcript list [25]. While APOD’s role in placental development has been relatively unexplored, existing reports suggest that reduced expression of APOD contributes to the development of placental hyperplasia diseases by inhibiting the migration and invasion of trophoblasts [44]. APOD is known to be involved in endosomal trafficking and internalization through a basigin (BSG, CD147) -dependent mechanism [45], which is also associated with the pathogenesis of PE [46]. The transmembrane glycoprotein basigin colocalizes with internalized APOD in vesicular compartments, and its down-regulation disrupts the internalization of APOD resulting PE. Additionally, APOD is implicated in modulating embryo-endometrium interactions, with increased mRNA expression in endometrium and epithelial cells (EEC) during the secretory phase after hormonal stimulation [20].

In normal pregnancy, spiral artery remodeling increases the arterial diameter by 5–tenfold [47], resulting in low-resistance, high-capacitance blood vessels that enhance blood delivery to the placenta [3]. This study demonstrates that overexpression of endometrial-gland derived APOD disrupts vascular remodeling by impairing EVT and endothelial cell functions in vitro, while APOD suppression enhanced these processes. These in vitro findings were corroborated in vivo using an endometrium-specific APOD knock-in mouse model, which recapitulated key ePE phenotypes including placental developmental abnormalities, key ePE phenotypes and elevated serum sFlt-1/PLGF levels [48, 49]. Collectively, these results indicate that elevated APOD disrupts placental development and contributes to PE pathogenesis, though the relative contributions of maternal versus other sources of circulating APOD warrant further investigation.

Mechanistically, this study demonstrates that APOD promotes placental ferroptosis through inhibition of the PI3K/AKT signaling pathway. PE is commonly associated with increased oxidative stress, resulting in the accumulation of ROS [50]. Ferroptosis, an iron-dependent form of cell death driven by lipid peroxidation, likely contributes to the pathogenesis of PE. In early pregnancy, alterations in placental oxygenation induce mitochondrial stress in trophoblasts [51], predisposing them to ferroptosis [52]. Trophoblasts are particularly susceptible to this process [53], which leads to macro-blebbing and vesiculation of the plasma membrane, oxidative damage, and disrupted angiogenic signaling. These changes ultimately result in trophoblast injury and placental dysfunction [52]. In PE patients, ferroptosis of trophoblasts may lead to incomplete spiral artery remodeling, causing placental malperfusion and oxidative stress, promoting further ferroptosis and forming a vicious cycle [54].

The regulatory mechanism of ferroptosis involves lipid, amino acid and iron metabolism. In PE, lipid peroxidation and accumulation of Fe^2+^ ions deplete intracellular antioxidants such as glutathione, triggering cell death via ferroptosis. GPX4 is the central regulatory molecule of ferroptosis, and decreased GPX4 activity reduces cellular antioxidant capacity and induces ferroptosis [55]. In human PE placentas, GPX4 levels are decreased [56, 57]. Multiple lines of evidence indicate the occurrence of ferroptosis in PE placentas, including disrupted mitochondrial outer membranes, reduced mitochondrial volume, loss of mitochondrial ridges [58, 59], increased placental malondialdehyde (MDA) [60], elevated placental Fe^2+^ [60], increased ROS [55, 59] and decreased placental superoxide dismutase (SOD) activity [61]. In this study, APOD inhibited PI3K, and lowered P-AKT levels and the P-AKT/AKT ratio in endometrium-specific APOD knock-in mice. Since the PI3K/AKT pathway is essential for enhancing angiogenesis [62] and suppression of ferroptosis [36, 37], these observations suggest that the APOD inhibits angiogenesis and spiral artery remodeling and induces ferroptosis in placenta via PI3K/Akt pathway in ePE.

In addition to its roles in lipid peroxidation and ferroptosis, APOD may also play a role in regulating endoplasmic reticulum (ER) stress, which is closely linked to ePE and PE [63–65, 97]. While there are no specific studies on APOD in this context, other research indicates that various apolipoproteins, such as APOE4, APOH, APOCIII, and APOB100, can induce ER stress, contributing to the development of various diseases. For instance, the human APOE4(1–272) fragment has been shown to trigger ER stress, as demonstrated by increased levels of ER stress markers in both in vivo and in vitro studies. This leads to tau hyperphosphorylation and disrupted axonal transport in Alzheimer’s disease [66]. Similarly, APOH has been found to induce ER stress during hepatitis B infection [67], APOB100 is associated with lipid-induced ER stress and hepatic insulin resistance [68], and APOCIII has been shown to cause ER stress and inflammation, while also impairing insulin signaling in mouse skeletal muscle cells [69]. These findings suggest that future research should explore the mechanisms by which APOD might contribute to the pathogenesis of PE through the modulation of ER stress.

Clinically, assessing multiple biomarkers can enhance the detection rate of PE [70]. Most existing PE biomarkers are based on the two-stage theory, which implicating poor placental perfusion to maternal endothelial dysfunction at around 12 weeks of gestation, thereby limiting their utility for early detection [48]. Since endometrial glandular activity peaks during the first trimester and insufficient glandular function may contribute to abnormal placental development and pregnancy failure [3, 8], glandular markers hold promise for earlier identification of abnormalities. Previous studies have also associated elevated levels of glycodelin, a major secretion of endometrial glands, with increased PE incidence and severity [15, 18], indicating that glandular products detectable in maternal serum could serve as early biomarkers. Our study demonstrated that maternal serum APOD is an accurate, reliable, and stable biomarker for early detection of PE. Unlike the widely used sFlt-1/PLGF ratio and patient characteristics [71–73], our findings, along with those of other studies [74], suggest that serum APOD can predict ePE from the first trimester and remains elevated in ePE patients compared to normotensive pregnant women throughout pregnancy. This supports its utility at multiple gestational stages. Current 1st-trimester screening combines maternal risk factors, mean arterial pressure, uterine artery Doppler, and biochemical marker such as PlGF achieves high detection for early-onset PE but more modest performance for later phenotypes of PE. Effective screening recommended by the Fetal Medicine Foundation (FMF) consists of a combination of maternal risk factors, mean arterial pressure, uterine artery pulsatility index (UtA-PI) and placental growth factor (PLGF). The current model has detection rates of 90%, 75%, and 41% for early, preterm, and term preeclampsia, respectively at 10% false-positive rate [93]. Compared to sFlt-1/PLGF, UtA-PI and PLGF, APOD may be detectable and informative earlier (11–13^+6^ weeks) potentially complementing existing tools [84, 85]. Moreover, as a single, stable plasma protein, APOD measurement is feasible within routine clinical chemistry laboratories, which may facilitate widespread implementation and reduce potential errors. Its concentration is minimally affected by gestational age, sampling time or maternal weight, yielding fewer false positives and curtailing repetitive monitoring and overtreatment. Given the clinical heterogeneity of PE, there remains a need to develop broader panels of early predictive biomarkers that reflect the pathophysiological processes of its different subtypes. The next stage will be to combine APOD with existing predictive indicators to see if this improves the overall detection rate of the current screening performance benchmark.

Based on our mouse model and clinical data, it is important to highlight that endometrial gland-derived APOD is a cause of PE, rather than a consequence of the condition. However, the underlying reasons for the increased glandular production of APOD in the endometrium during pregnancy complicated by PE remains unknown. Increasing evidence has associated aberrant decidualization with the occurrence of PE [75, 76], which may adversely affect the bi-directional signalling between the stromal and gland cells essential for normal placentation [8, 14]. Another potential mechanism may involve epigenetic dysregulation associated with PE [77]. For example, various genes exhibit differential methylation in the placenta and endothelial cells of women with PE [78]. DNA hypomethylation of PlGF and sFlt-1, and subsequent their changes in gene expression have also been observed in women with PE [79]. APOD DNA/RNA methylation has been implicated in the pathogenesis of gastric cancer [80, 81], and so studying APOD methylation in PE could provide valuable insights. Most accounts attribute the cause of ePE to deficient remodelling of the spiral arteries secondary to abnormal interactions among extravillous trophoblasts, fetal antigens and uNK cells [86–88]. Our organoid and mouse data suggest an upstream glandular factor-APOD can impair EVT/endothelial functions, potentially interacting with immune pathways. APOD is localized primarily to glands and that gland-derived factors can diffuse to regions where uNK-EVT interactions occur in our stereo-seq analysis (Figure S12). In future work we will map gland-APOD signaling to EVT and uNK cells in early decidua using spatial multi-omics, and model how secreted APOD remotely steers their crosstalk.

The use of endometrial glands obtained at term from PE patients, rather than from first-trimester tissues, is primarily due to ethical and logistical challenges associated with accessing early pregnancy samples. Although the endometrial environments differ between term and early pregnancy, recent studies have shown that endometrial epithelial cells cultured as organoids display significant cellular plasticity. Under appropriate hormonal and culture conditions, these organoids can recapitulate key features of the early pregnancy endometrium and the disease phenotype observed in vivo, similar to organoids derived from other organs [82, 83]. Nonetheless, we recognize that future studies utilizing first-trimester tissues will be essential for further validation. Additionally, the apical-in/basal-out polarity of endometrial organoids means that conditioned medium contains mixed basal and diffused apical secretions. Deriving apical-out organoids that retain the in vivo polarity would generate more physiological secretions.

Another limitation of this study is that pre-eclampsia (PE) is unique to humans and does not occur in mice. However, our endometrial-specific APOD knock-in mouse model enabled us to investigate placental development mechanisms that cannot be ethically studied in humans. We acknowledge that Tie2-Cre-mediated recombination can occur in both endothelial and certain non-endothelial compartments, including hematopoietic progenitors, which should be considered when interpreting our conditional knockout findings. To further address this limitation, future research can utilize human trophoblast stem cell–derived organoids to explore how endometrial gland secretions influence trophoblast lineage decisions [95]. In addition, APOD should be integrated into first-trimester PE prediction algorithms and validated in larger cohorts to assess whether maternal serum APOD improves existing models that include mean arterial pressure (MAP), uterine artery pulsatility index (UtA-PI), and PlGF/sFlt-1. Finally, our spatial transcriptomics analysis was conducted on full-thickness endometrium from the non-pregnant secretory phase, which lacks endovascular cytotrophoblasts. Future studies should extend these analyses to pregnant decidua to elucidate the spatiotemporal interactions among gland-derived signals, endovascular extravillous trophoblasts (EVTs), and uterine natural killer (uNK) cell niches, thereby providing a more comprehensive understanding of endometrial physiology and pathological remodeling.

## Conclusions

**Fig. 6:**
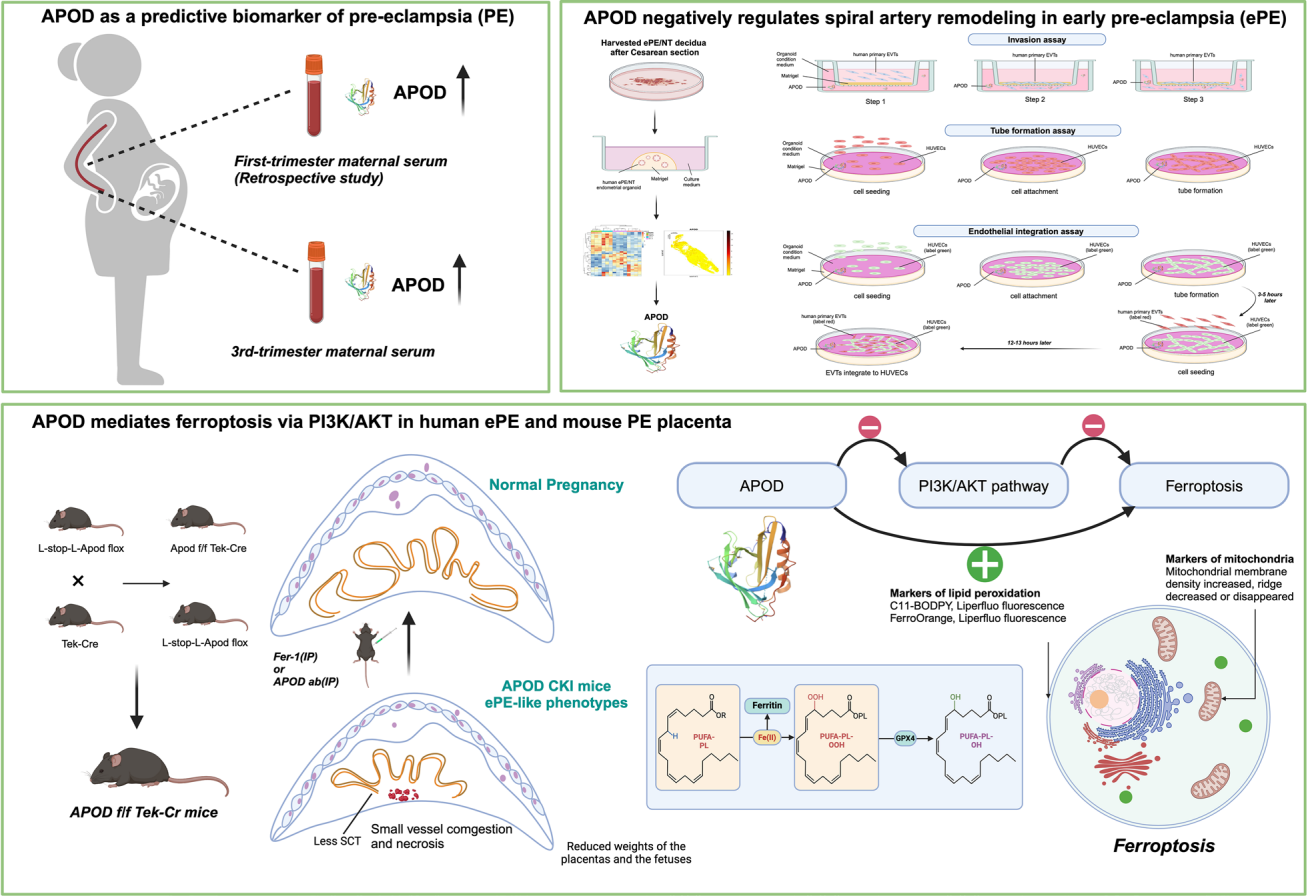
Dysregulated endometrial gland-derived APOD is a maternal contributor to early-onset pre-eclampsia. Dysregulated endometrial gland-derived APOD is a maternal contributor to early-onset pre-eclampsia (ePE). Elevated APOD levels in both first and third trimester maternal serum support its potential as a predictive biomarker for ePE. Excess APOD impairs spiral artery remodeling during early pregnancy by inhibiting extravillous trophoblast (EVT) invasion, endothelial cell angiogenesis, and EVT-endothelial cell integration. Endometrial-specific APOD knock-in in pregnant mice induced an ePE-like phenotype, characterized by reduced syncytiotrophoblast (SCT) formation, small vessel congestion and necrosis, and decreased placental and fetal weights. Mechanistically, APOD promotes placental ferroptosis by inhibiting the PI3K/AKT pathway. Treatment with the ferroptosis inhibitor Fer-1 alleviated placental defects and ePE symptoms in APOD knock-in mice

In summary, this study demonstrates that dysregulated endometrial gland-derived APOD is a maternal contributor to early-onset pre-eclampsia (Fig. 6). Through inhibiting spiral artery remodeling and promoting placental ferroptosis via the PI3K/AKT pathway, elevated APOD disrupts trophoblast integration and leads to insufficient placental development. It may also induce placental ER stress that contributes to the relase of pro-inflammatory cytokines. Moreover, its increased concentration in early pregnancy serum underscores APOD’s potential value as an early predictive biomarker and therapeutic target for ePE by interfering APOD-induced ferroptosis.

## Supplementary Information

Supplementary Material 1.

Supplementary Material 2.

Supplementary Material 3.

Supplementary Material 4.

Supplementary Material 5.

## Data Availability

The mRNA-seq data generated in this study have been deposited in the NCBI GEO database under accession code GSE287645. Spatial-seq data are available at the National Genomics Data Center (NGDC) under accession number OMIX009055. Additional datasets and material requests should addresd to the corresponding author.

## Abbreviations

ePE: Early-onset pre-eclampsia
APOD: Apolipoprotein D
PE: Pre-eclampsia
FGR: Fetal growth restriction
EVT: Extravillous trophoblast
GOS: Great Obstetrical Syndrome
FOXA2: Forkhead Box A2
LIF: Leukemia inhibitory factor
NT: Normotensive
PAX8: Paired Box Gene 8
SOX17: SRY-Box Transcription Factor 17
DAB: Diaminobenzidine
DNB: DNA nanoballs
GO: Gene Ontology
KEGG: Kyoto Encyclopedia of Genes and Genomes
CID: Coordinate identity
UMIs: Unique molecular identifiers
DDA: Data-dependent acquisition
DEPs: Differentially expressed proteins
TMB: 3,3′,5,5′-Tetramethylbenzidine
HUVECs: Human Umbilical Vein Endothelial Cells
Fer-1: Ferroptosis inhibitor
PFA: Paraformaldehyde
TEM: Transmission electron microscope
HBSS: Hanks’ Balanced Salt Solution
DEGs: Differentially expressed mRNAs
PCA: Principal component analysis
iTRAQ: Isobaric tags for relative and absolute quantitation
ELISA: Enzyme-linked immunosorbent assay
sFlt-1: Soluble fms-like tyrosine kinase-1
PLGF: Placental growth factor
TPBPA: Trophoblast-specific protein alpha
MCT4: Monocarboxylate transporter 4
P-AKT: Phosphorylated AKT
MDA: Malondialdehyde
SOD: Superoxide dismutase
UGKO: Endometrial gland knockout
EEC: Epithelial cells
ROS: Reactive oxygen species
ER: Endoplasmic reticulum
