# Supplementary material for: Maternal causation of early-onset pre-eclampsia: excessive endometrial gland-derived apolipoprotein D induces placental ferroptosis and developmental abnormalities
Source: J Biomed Sci. 2025 Dec 10;32:103. doi: 10.1186/s12929-025-01199-7 (PMC12690863; doi:10.1186/s12929-025-01199-7)
Supplement: Supplementary file 1 — Supplementary Material 1. [file 12929_2025_1199_MOESM1_ESM.pdf]

Figure S1. Schematic diagram for establishment of primary human endometrial organoids, related to Figure 1.

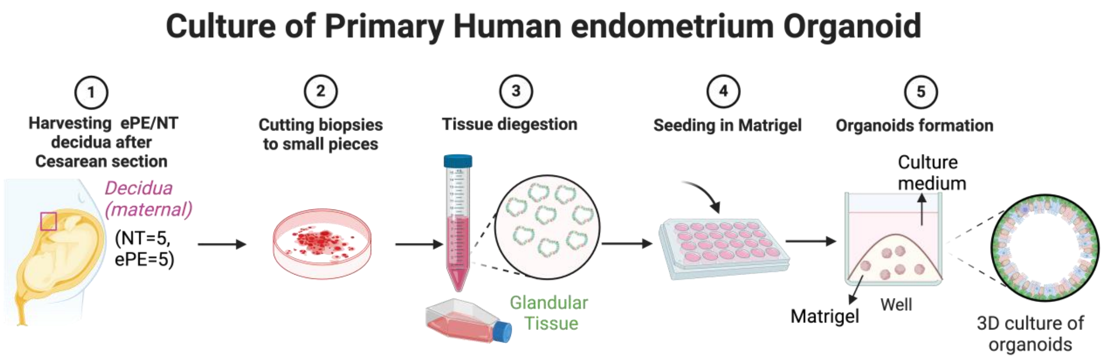

Figure S1 Schematic diagram for establishment of primary human endometrial organoids. Schematic diagram showing the establishment of primary human endometrial organoids derived from normotensive (NT) and early-onset pre-eclampsia (ePE) pregnancies.

Figure S2. Primary extravillous trophoblast (EVTs) isolated from first trimester placental villi, related to Figure 1.

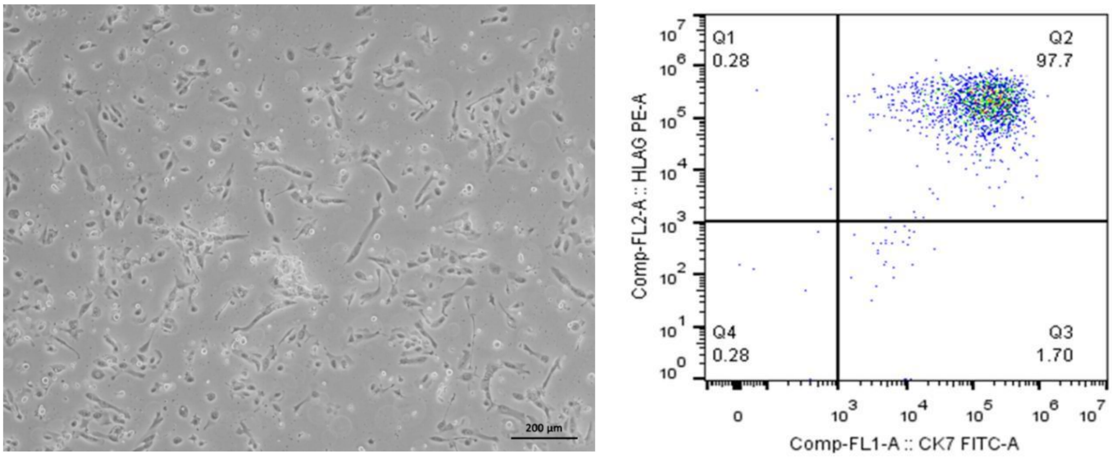

Figure S2 Primary extravillous trophoblast (EVTs) isolated from first trimester placental villi. EVTs were isolated from first-trimester placental villi were analyzed by flow cytometry using CK7 (pan trophoblast marker) and HLAG (EVT marker).

Figure S3. ePE and NT organoid secretome didn't affect HUVECs/EVTs viability, related to Figure 1.

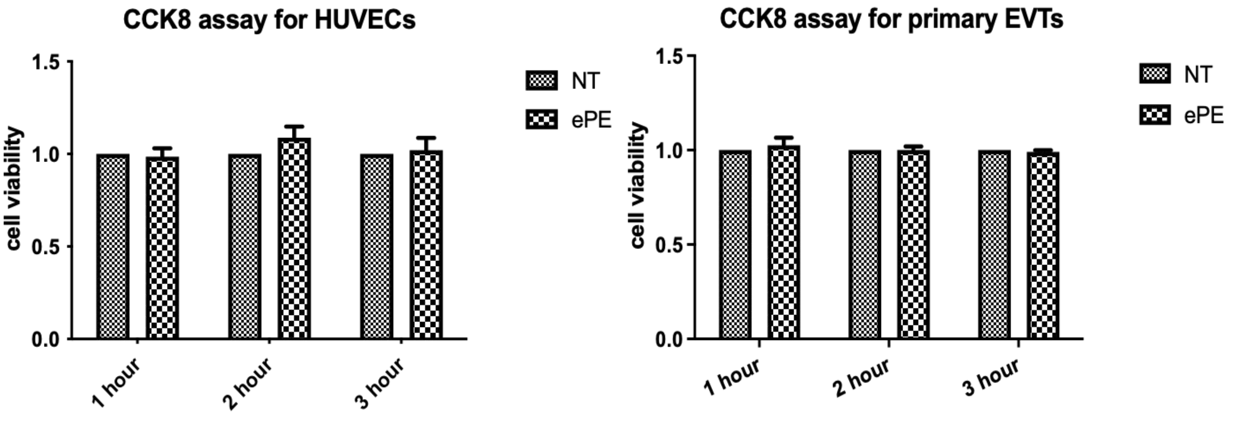

Figure S3 ePE and NT organoid secretome did not affect HUVECs/EVTs viability.

HUVECs or EVTs ($5x10^3$ cells/well) were cultured with 100μL organoid conditioned medium in 96-well plates for 24 h. Absorbance at 450 nm was measured after incubating the cells with 10μL sterile CCK8 dye for 1, 2 and 3 h at 37°C. Cell viability (%) was calculated using the formula: (Absorbance of Test- Blank Absorbance)/ (Absorbance of Control- Blank Absorbance) x100%. All the data are expressed as mean ± SD. N=3.

Figure S4. KEGG pathway analysis and GO analysis of DEG, related to Figure 1.

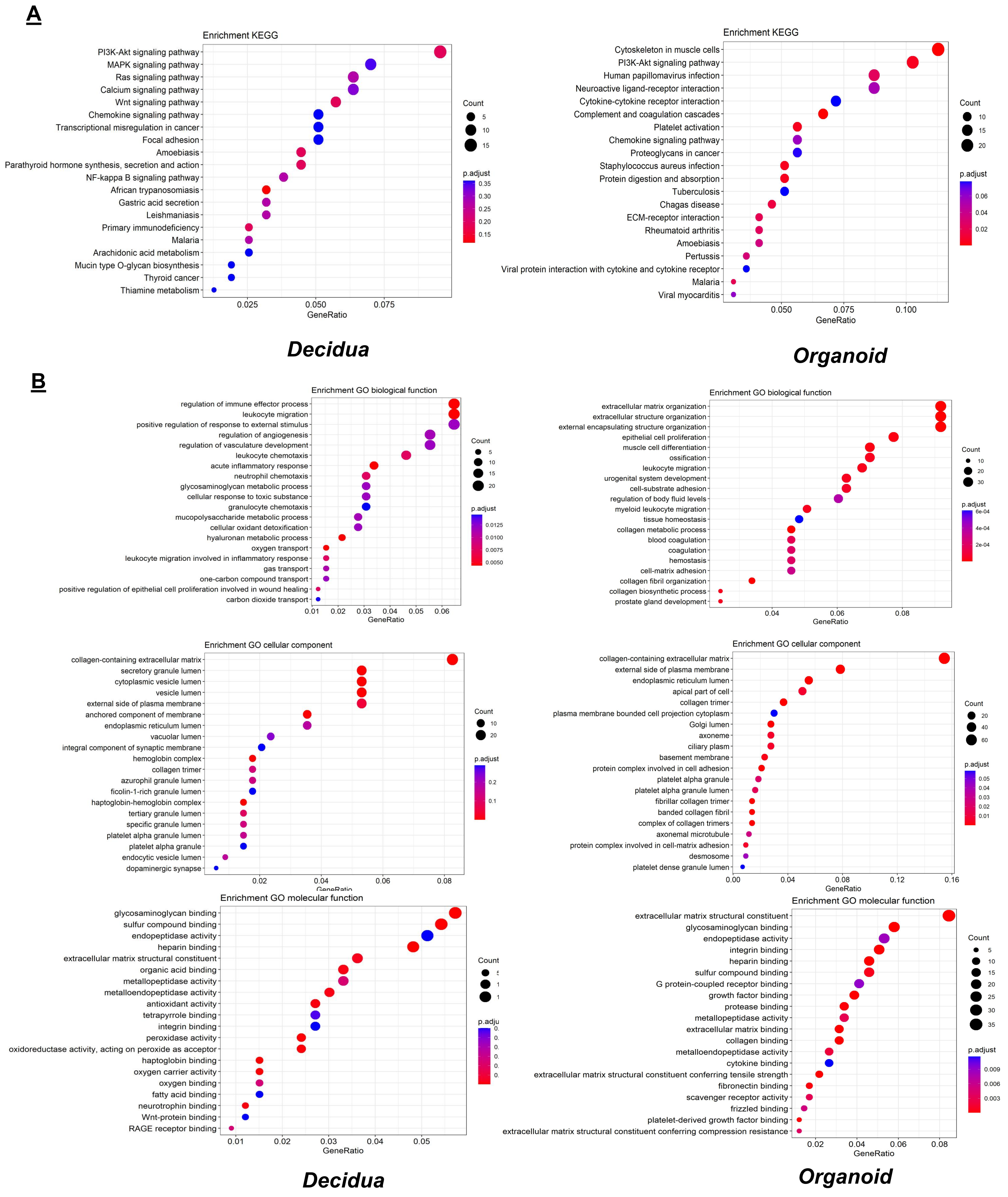

Figure S4 KEGG pathway analysis and GO analysis of DEG.

(A) KEGG pathway analysis of differentially expressed mRNA genes to identify enriched signaling pathways. **(B)** Gene Ontology (GO) analysis of differentially expressed mRNA genes. Human decidua tissue, N=3. Endometrial organoids, N=4.

Figure S5. KEGG pathway analysis and GO analysis of DEP, related to Figure 1.

A

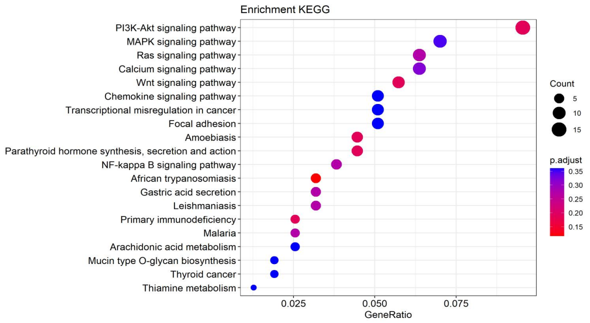

B

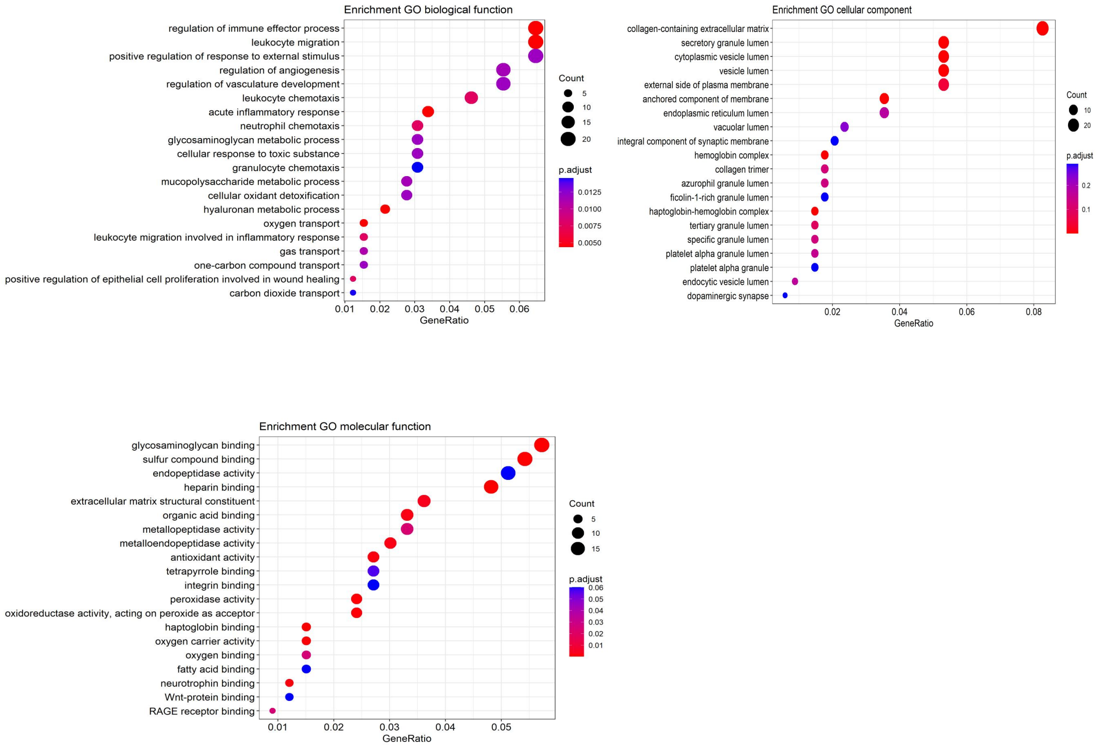

Figure S5 KEGG pathway analysis and GO analysis of DEP.

(A) KEGG pathway analysis and **(B)** GO analysis of DEP. N=5.

Figure S6. Lentiviruses transfected into NT and ePE organoids, related to Figure 2.

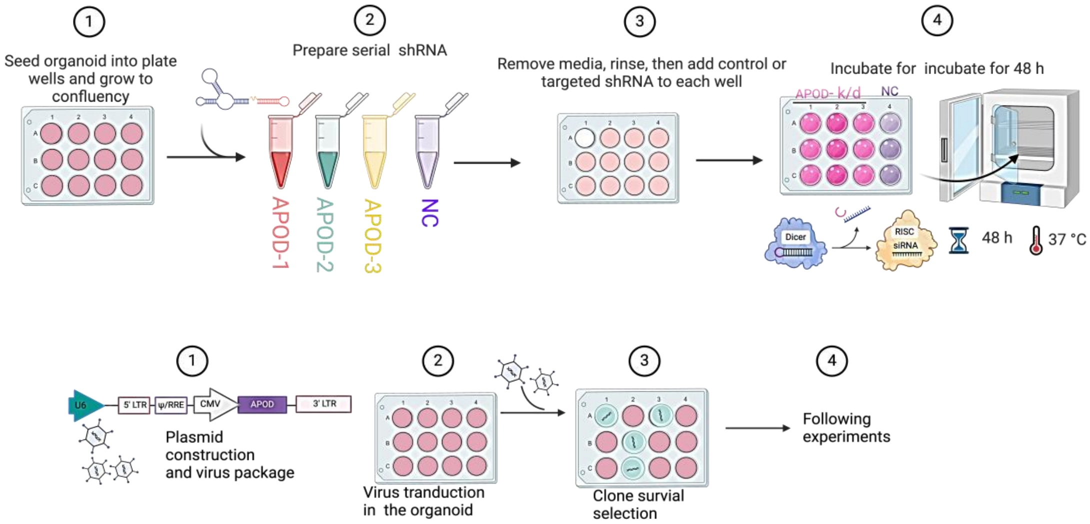

Figure S6 Lentiviruses transfected into NT and ePE organoids.

Overexpression (20.8×10^6 Tu/well) and knockdown (12.5×10^6 Tu/well) lentiviruses were transfected into NT and ePE organoids, respectively, using Hitrans G-B-2.

Figure S7. Lentivirus did not affect HUVECs/EVTs viability, related to Figure 2.

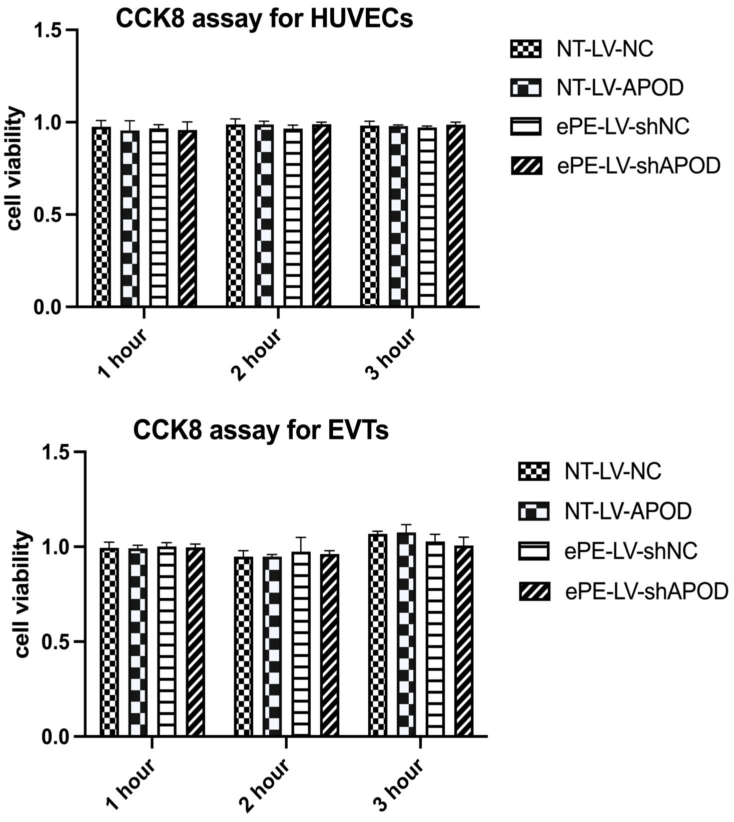

Figure S7 Lentivirus did not affect HUVECs/EVTs viability.

HUVECs or EVTs ($5x10^3$ cells/well) were cultured with 100μL organoid conditioned medium after lentivirus infection in 96-well plates for 24 h. Absorbance at 450 nm was measured after incubating the cells with 10μL sterile CCK8 dye for 1, 2 and 3 h at 37°C. Cell viability was calculated using the formula: (Absorbance of Test- Blank Absorbance)/ (Absorbance of Control- Blank Absorbance) x100%. All the data are expressed as mean ± SD. N=3.

Figure S8. APOD antibody did not affect HUVECs/EVTs viability, related to Figure 2.

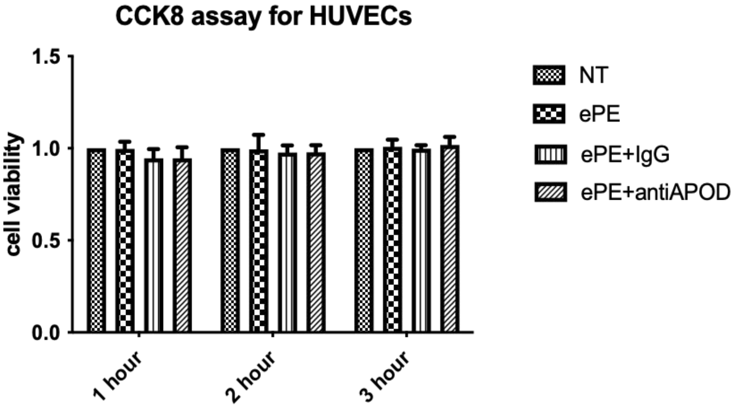

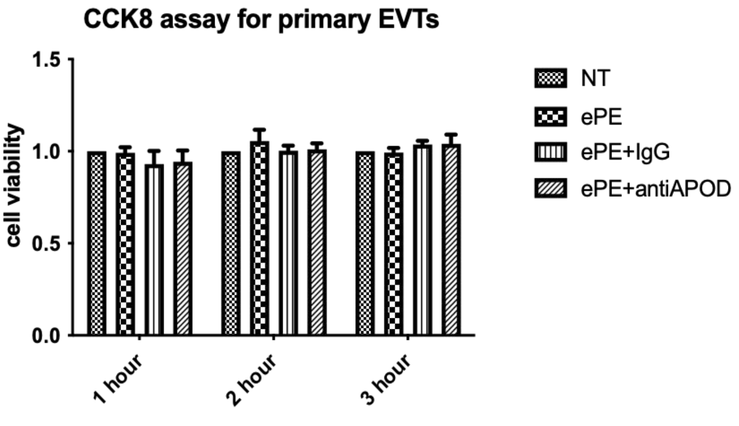

Figure S8 APOD antibody did not affect HUVECs/EVTs viability.

HUVECs or EVTs (5x10^3 cells/well) were cultured with 100μL organoid conditioned medium with/without blocking anti-APOD antibody (1:300) in 96-well plates for 24 h. Absorbance at 450 nm was measured after incubating the cells with 10μL sterile CCK8 dye for 1, 2 and 3 h at 37°C. Cell viability was calculated using the formula: (Absorbance of Test- Blank Absorbance)/ (Absorbance of Control- Blank Absorbance) x100%. All the data are expressed as mean ± SD. N=3.

Figure S9. APOD did not affect the number of pups, related to Figure 3.

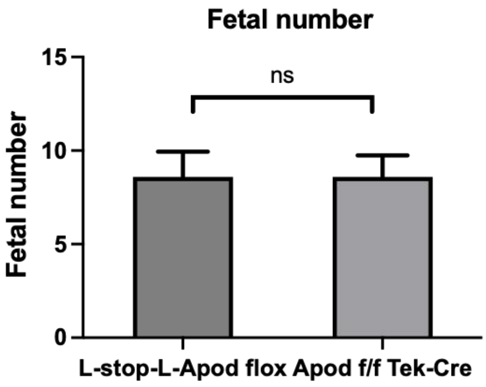

Figure S9 APOD did not affect the number of pups.
Pups number of L-stop-L-Apod flox and Apod f/f Tek-Cr pregnant mice. N = 5.

Figure S10. APOD didn't affect pregnant mice weight from E11.5 to E17.5, related to Figure 3.

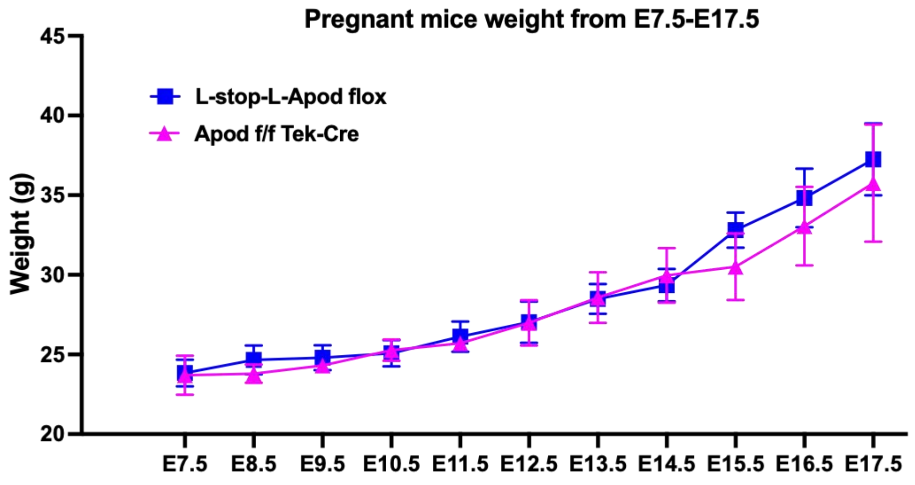

Figure S10 APOD did not affect pregnant mice weight from E11.5 to E17.5 during pregnancy. Weight of L-stop-L-Apod flox and Apod f/f Tek-Cr pregnant mice from E7.5 to E17.5. N = 5.

Figure S11. Activator/inhibitor studies suggested ePE-organoids secretome impairs EVT invasion via PI3K–AKT pathway, related to Figure 4.

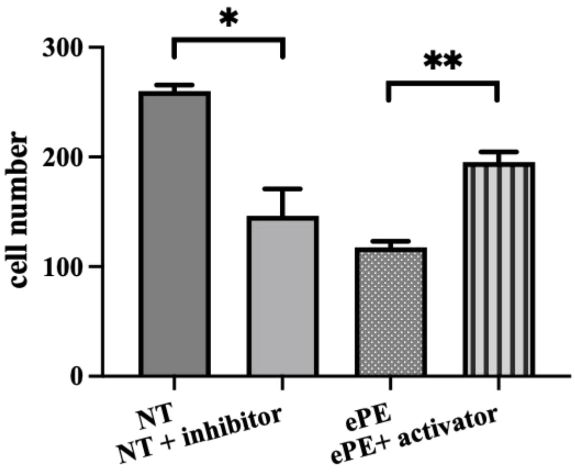

Figure S11 For invasion assay, EVTs (1 × 10^5 cells/well) were seeded into the upper chamber of Matrigel-coated Transwell inserts. The lower chamber was filled with 600 µL of organoid-conditioned media with the PI3K-akt activator (HY-151527, MCE, USA) 10µM and inhibitor (HY-144806, MCE, USA) 1µM. N=3. Data are expressed as mean ± SD. *$p < 0.05$, **$p < 0.01$.

Figure S12. The expression of uNK in Spatial transcriptome profiling of full-thickness endometrium, related to Discussion.

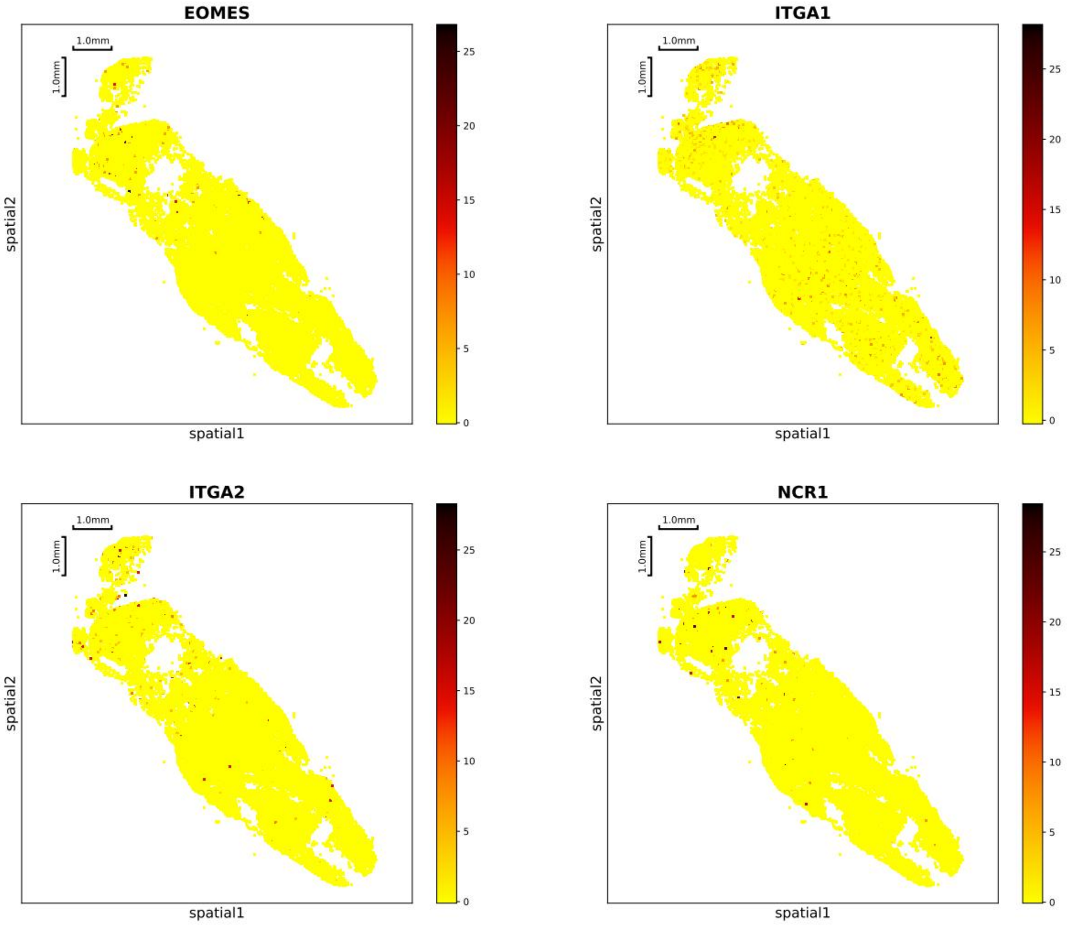

Figure S12 The expression of uNK (markers: EOMES, ITGA1, ITGA2, NCR1) are located near the gland in Spatial transcriptome profiling of full thickness human endometrium.
