# Supplementary material for: Maternal causation of early-onset pre-eclampsia: excessive endometrial gland-derived apolipoprotein D induces placental ferroptosis and developmental abnormalities
Source: J Biomed Sci. 2025 Dec 10;32:103. doi: 10.1186/s12929-025-01199-7 (PMC12690863; doi:10.1186/s12929-025-01199-7)
Supplement: Supplementary file 2 — Supplementary Material 2. [file 12929_2025_1199_MOESM2_ESM.pdf]

Table S1. Patient information on endometrial specimens for constructing endometrial glandular organoids, related to Figure 1.

Human decidua tissue (28-42 weeks of gestation)			
	ePE group (N=5)	NT group (N=5)	P value
Age at EDC (years)	31.0±5.8	30.0±4.7	0.773
Gest weeks (weeks)	32.2±1.7	39.2±1.3	0.000
Maternal weight (kg)	70.7±7.0	65.2±9.2	0.317
Systolic pressure (mmHg)	165 ±8	115±7	0.000
Diastolic pressure (mmHg)	108±11	79±6	0.001
Proteinuria (+)	2.6±1.1	0.2±0.5	0.002
Fetal Birth Weight (g)	1527±322	3684±411	0.000
Placental Weight (g)	351±40	560±29	0.000

Table S4. Patient information for verify APOD expression in serum during early pregnancy, related to Figure 5.

Maternal serum sample (11-13^{+6} weeks of gestation)			
	PE group (N=14)	NT group (N=14)	P value
Age at EDC (years)	35.1±4.8	35.1±4.8	0.969
Gest weeks (weeks)	37.2±1.8	38.2±1.5	0.116
Maternal weight (kg)	64.2±11.3	56.2±9.9	0.059
Systolic pressure (mmHg)	151±9	114±11	0.000
Diastolic pressure (mmHg)	96±8	72±9	0.000
Proteinuria (+)	1.3±0.6	0.0±0.0	0.000
Fetal Birth Weight (g)	2590±480	2890±488	0.113
Placental Weight (g)	492±101	605±172	0.042

Table S5. Patient information for verify APOD expression in serum during late pregnancy, related to Figure 5.

Maternal serum sample (28-42 weeks of gestation)			
	PE group (N=15)	NT group (N=15)	P value
Age at EDC (years)	31.9±3.7	31.6±5.2	0.841
Gest weeks (weeks)	35.1±3.2	39.2±0.6	0.000
Maternal weight (kg)	63.1±7.9	63.4±7.1	0.914
Systolic pressure (mmHg)	163±9	115±7	0.000
Diastolic pressure (mmHg)	101±15	71±9	0.000
Proteinuria (+)	2.1±1.1	0.1±0.4	0.000
Fetal Birth Weight (g)	2656±724	3664±215	0.000
Placental Weight (g)	442±80	574±47	0.000

Table S6. Organoid medium components, related to Methods.

Product	Company	Product Number	Final Concentration
Advanced DMEM/F12	Life Technologies	12634010	1X
N2 supplement	Life Technologies	17502048	1X
B27 supplement minus vitamin A	Life Technologies	12587010	1X
Nicotinamide	Sigma	N0636	10 nM
N-Acetyl-L-cysteine	Sigma	A9165-5G	1.25 mM
L-glutamine	Life Technologies	25030-024	2 mM
Recombinant human EGF	Peprotech	AF-100-15	50 ng/ml
Recombinant human Noggin	Peprotech	120-10c	100 ng/ml
Recombinant human Rspondin-1	Peprotech	120-38	500 ng/ml
Recombinant human FGF-10	Peprotech	100-26	100 ng/ml
Recombinant human HGF	Peprotech	100-39	50 ng/ml
ALK-4,-5,-7 inhibitor, A83-01	Merck	688000	500 nM
Primocin	Invivogen	ant-pm-1	100 u g/ml

Table S7. Summary of all antibodies used in this study, related to Methods.

name	species	clone name	cat no.	company	dilution
FOXA2	Human, Mouse	Rabbit Monoclonal [EPR4466]	ab108422	Abcam	1:100
PAX8	Human	Rabbit Monoclonal [SP348]	ab227707	Abcam	1:100
Isotype Control	Human	Rabbit Monoclonal [EPR25A]	ab199376	Abcam	1:100
APOD	Human	Rabbit Monoclonal [EPR2916]	ab108191	Abcam	1:300
APOD	Human, Mouse, Rat	Rabbit Polyclonal	PA527386	Thermofisher	1:100
MCT4	Mouse, Rat, Human	Rabbit Monoclonal [EPR28177-30]	ab308528	Abcam	1:100
Tpbpa	Mouse	Rabbit Monoclonal	ab320823	Abcam	1:100
GPX4	Human, Mouse, Rat	Rabbit Polyclonal	#30388-1-AP	proteintech	1:100
Alexa Fluor 647	Rabbit	Donkey Polyclonal	A-31573	Invitrogen	1:400
Alexa Fluor 488	Rabbit	Donkey Polyclonal	A-21206	Invitrogen	1:400
PI3K	Human, Mouse, Rat, Bovine	Rabbit Monoclonal	#4249	Cell Signaling	1：1000
P-AKT	Human, Mouse, Rat, Hamster, Monkey, D.Melanogaster, Zebrafish, Bovine	Rabbit Monoclonal	#4060	Cell Signaling	1：1000
AKT	Human, Mouse, Rat, Monkey, D.Melanogaster,	Rabbit Monoclonal	#4691	Cell Signaling	1：1000
HRP-linked Antibody	Rabbit	Goat Polyclonal	#7074s	Cell Signaling	1：1000

Table S8. Three shRNAs designed to reduce APOD in PE organoids, related to Methods.

GENE & NO.	Target Seq
APOD-RNAi (1)	gcCACCGACTATGAGAACTAT
APOD-RNAi (2)	cgGAAAGATCAAAGTGTTAAA
APOD-RNAi (3)	tgAATAAGTATCTCGGAAGAT

Table S9. The steps for removing genomic DNA, related to Methods.

Reagent	Volume (µl)
5×gDNA Eraser Buffer	2.0
gDNA Eraser	1.0
Total RNA	1.0
RNase Free ddH_2O	Up to 10

Table S10. mRNA reverse transcription system, related to Methods.

Reagent	Volume (μl)
Step 1 Solution (table 3)	10.0
PrimeScript RT Enzyme Mix	1.0
RT Primer Mix	1.0
5×PrimeScript Buffer 2 (for Real Time)	4.0
RNase Free ddH_2O	4.0
Total	20

Table S11. Primers and other short nucleotide sequences used in the study, related to Methods.

Gene Name		Sequence (5'to 3')
APOD	Forward	GCT GGA AGT TAA GTT TTC CTG G
	Reverse	GGA TGA TGC AGG TAC AGG AAT A
uPA	Forward	AATTTCAGTGTGGCCAAAAGAC
	Reverse	GTCCTCCTTCTTTGGGTAATCA

Table S12. SYBR Green Real-time PCR amplification reaction system, related to Methods.

Reagent	Volume (µl)
2*Taq Pro Universal SYBR qPCR Msater Mix	10.0
PCR Forward Primer (10 µm)	0.4
PCR Reverse Primer (10 µm)	0.4
CDNA	2.0
ddH_2O	7.2
Total	20
